# Prognostic immunological implications of OX40L expression in the tumor microenvironment of melanoma

**DOI:** 10.3389/fimmu.2026.1745742

**Published:** 2026-01-23

**Authors:** Susanna Feldman, Baseem Da’ana, Nona Ofek, Liliana Elkis, Adi Pollak-Moseri, Emmanuela Riklin-Nahmias, Sonia Mendlovic, Ayelet Avraham, Raya Leibowitz

**Affiliations:** 1 Department of Oncology, Shamir Medical center, Beer Yaakov, Israel; 2 Department of Pathology and Laboratory Medicine LabPlus, Auckland, New Zealand; 3 Department of Pathology, Barzilai Medical Center, Ashkelon, Israel; 4 Department of Pathology, Shamir Medical center, Beer Yaakov, Israel; 5 Gray Faculty of Medical and Health sciences, Tel-Aviv University, Tel Aviv, Israel

**Keywords:** melanoma, OX40L (TNFSF4), recurrence/prognosis, regulatory T cells (Treg), tumor microenvironment (TME)

## Abstract

**Background:**

Immunotherapy targeting immune checkpoint proteins (ICPs) has transformed cancer care, yet current treatments focus on a narrow set of inhibitory ICPs and benefit only a subset of patients. The co-stimulatory pair OX40–OX40L, implicated in inflammation and autoimmunity, also plays roles in cancer immunity. We previously showed that high OX40L mRNA expression in melanoma correlates with favorable prognosis and improved responses to PD-1 blockade. However, the protein-level expression and functions of OX40L in melanoma remain poorly defined.

**Methods:**

Formalin-fixed paraffin-embedded primary tumor samples from 30 patients with stage II–III melanoma were analyzed by multiplex immunofluorescence combined with quantitative image analysis. OX40L and OX40 expression were evaluated alongside immune cell phenotyping markers. Regulatory T cells (Tregs) isolated from human peripheral blood were examined by flow cytometry and RT-qPCR. Associations with recurrence were assessed in depth-matched subsets (n=22) using Kaplan–Meier analysis.

**Results:**

OX40L was detected across tumor, immune, and stromal compartments, with marked intertumoral heterogeneity. OX40^+^ cells were less frequent but were often found in spatial proximity to OX40L^+^ cells. OX40L was infrequently detected in melanoma cancer cells, and was more prevalent in antigen-presenting cells, CD4^+^/CD8^+^ T cells, and regulatory T cells. Strikingly, intratumoral Tregs expressed OX40L more frequently than OX40 or other ICPs, whereas blood-derived Tregs showed the opposite pattern, with OX40 predominating over OX40L. Disease recurrence following resection of the primary tumor was associated with lower proportions of OX40L-expressing myeloid cells, providing preliminary evidence for a potential link between myeloid OX40L expression and recurrence risk.

**Conclusions:**

OX40L protein expression is a heterogeneous but prominent feature of the melanoma microenvironment, with cell type–specific expression patterns that include regulatory T cells. An exploratory association was seen between myeloid OX40L expression and clinical outcome, warranting further investigation.

## Introduction

The discovery of immune checkpoint proteins (ICPs) and the development of monoclonal antibodies targeting them have enabled robust and reproducible activation of the immune system against cancer (1). Currently approved immune checkpoint inhibitors (ICIs) act on inhibitory ICPs at the cancer–immune synapse, including PD-1/PD-L1, CTLA-4, and LAG-3. Despite major clinical success, these therapies benefit only subsets of patients across select malignancies and seldom achieve durable cure. This has prompted efforts to expand immunotherapeutic strategies toward additional targets at the cancer-immune interface–often designated ‘the immunological synapse’–encompassing both inhibitory and co-stimulatory pathways (2).

One such co-stimulatory pair is OX40 and its ligand OX40L, members of the tumor necrosis factor superfamily, also designated TNFRSF4 and TNFSF4, respectively. OX40 is induced on activated—but not resting—T cells, where it delivers co-stimulatory signals that enhance effector activity, prolong T-cell responses, and promote memory formation. Its ligand, OX40L, is preferentially expressed on antigen-presenting cells (APCs) such as B cells, dendritic cells, and macrophages, but is also found on other cell types including Langerhans cells, endothelial cells, mast cells, and natural killer (NK) cells. This distribution suggests that OX40–OX40L interactions shape diverse physiological responses between T cells and both immune and non-immune compartments (3, 4). Consistent with these roles, OX40–OX40L signaling is centrally involved in inflammatory and autoimmune diseases (5).

In cancer, OX40 agonist antibodies or OX40L-based gene constructs have produced robust anti-tumor immune responses and tumor regression in animal models (6). However, early-phase clinical trials employing these strategies have so far yielded limited efficacy, though combinations with ICIs are actively under investigation (reviewed in (6); clinicaltrials.gov IDs NCT03323398, NCT03739931). We previously analyzed RNA-sequencing data from The Cancer Genome Atlas (TCGA) and observed that low OX40L expression correlated with poorer prognosis across melanoma stages. Moreover, metastatic melanoma patients with low OX40L mRNA had significantly worse outcomes and reduced response rates to anti–PD-1 therapy (7). However, evidence of OX40L protein expression in melanoma tumors is still lacking, and further studies are required to delineate the role of OX40L in the tumor microenvironment (TME). To address this gap, we conducted an in-depth analysis of OX40L protein patterns in primary melanoma, aiming to study their distribution across tumor and immune compartments and their potential impact on immune regulation within the tumor microenvironment (TME).

## Materials and Methods

### Patients and tissue specimens

Samples of primary tumors from 30 patients with stage I–III cutaneous melanoma who underwent biopsy or resection at Shamir Medical Center (2016–2023) were analyzed following IRB approval. Patients with prior systemic therapy or non-cutaneous melanoma were excluded. Clinicopathologic features and follow-up data (median 43 months, range 12–92) are summarized in **Supplementary Table S1**.

### Multiplex immunofluorescence and image analysis

Multiplex immunofluorescence (mIF) was performed on FFPE sections using sequential tyramide signal amplification (Tyramide SuperBoost kit, Thermo Fisher Scientific, Waltham, MA, USA). Slides were counterstained with DAPI to visualize all nucleated cells and mounted in EverBrite™ Hardset medium (Biotium, Fremont, CA, USA).

Regions of interest (ROIs) were manually selected, and images were acquired using a Biotek Lionheart FX microscope (Agilent, Santa Clara, CA, USA). Quantitative analysis was performed with GEN5 Prime software (Agilent) for automated cell segmentation and cell counting, while autofluorescent structures and normal skin elements with physiological OX40L expression, such as vessels, were manually excluded. Distinct distance thresholds were applied to differentiate marker co-expression within the same cell (<2 µm) from spatial proximity between different cells (<20 µm). Other quantification, co-expression, and proximity analyses are detailed in **Supplementary Methods** and **Supplementary Table S2**. Anti-OX40L antibody specificity was validated using melanoma cell lines stably overexpressing OX40L and matched negative controls, with confirmation of expression at the mRNA and protein levels and by immunofluorescence in FFPE-processed cell blocks, as detailed in **Supplementary Methods** and **Supplementary Figure S7**.

### Treg isolation and expansion

Peripheral blood mononuclear cells (PBMCs) were isolated from fresh buffy coat obtained from an anonymous healthy donor (Blood Bank Laboratories, Sheba Medical Center, Tel Hashomer, Israel) by density-gradient centrifugation. Tregs were enriched using a CD4^+^CD25^+^CD127^-^ selection kit (EasySep™, STEMCELL Technologies, Vancouver, Canada), yielding ~1.6 × 10^6^ Tregs per batch from 5 × 10^7^ PBMCs; three independent batches were processed. Post-enrichment, 80–90% of viable cells were confirmed as CD4^+^CD25^+^ by flow cytometry.

Tregs were expanded in ImmunoCult™-XF medium (STEMCELL Technologies) supplemented with recombinant human IL-2 (Sigma-Aldrich, St. Louis, MO, USA) and antibiotics (Biowest, Nuaillé, France). Cells were stimulated with CD3/CD28 activator (STEMCELL Technologies) on days 0 and 7. Expansion was monitored by cell counts and viability.

### Flow cytometry

Cells were stained with fluorophore-conjugated antibodies against CD4, CD25, OX40, OX40L, and intracellular Foxp3 using the Foxp3 Buffer Set (Miltenyi Biotec, Bergisch Gladbach, Germany). Cell viability was assessed using Viobility™ dye (Miltenyi Biotec). Data acquisition and analysis followed a predefined sequential gating strategy. Briefly, cells were first gated on forward scatter (FSC-A) and side scatter (SSC-A) to exclude debris, followed by singlet discrimination using FSC-A versus FSC-H. Live cells were identified by exclusion of Viobility™-positive events. Regulatory T cells were then defined as Foxp3^+^ cells within the live singlet population, and subsequent analyses were performed by gating Foxp3^+^ cells for expression of OX40L versus OX40 or CD25 versus CD4, as indicated.

Gating thresholds for each marker were defined individually using unstained control samples freshly prepared at each experimental time point. Data were acquired on a MACSQuant^®^ Analyzer 10 and analyzed using MACSQuantify™ software v3.02 (Miltenyi Biotec). Spectral overlap was compensated using single-stained antibody controls and pre-stained compensation beads (Miltenyi Biotec). Antibody details and fluorophore combinations are provided in **Supplementary Table S3**.

### RNA extraction and RT-qPCR

RNA was extracted with the SV Total RNA kit (Promega, Madison, WI, USA). Purity was confirmed by NanoDrop (Thermo Fisher Scientific). cDNA synthesis was performed with the SuperScript III First-Strand kit (Thermo Fisher Scientific), and qPCR was run in duplicate using PerfeCTa SYBR Green FastMix (Quantabio, Beverly, MA, USA) on a Rotor-Gene 6000 instrument (Corbett, Sydney, Australia). Expression was normalized to GAPDH, β-actin, and TFRC. Primer sequences are listed in **Supplementary Table S4**.

### Statistical analysis

Quantitative data are presented in box plots displaying means, medians, and interquartile ranges to account for non-normal distributions and potential outliers. Comparisons between groups were performed using two-tailed Student’s *t*-tests applied to ROI-level data (8–15 ROIs per tumor) to assess differences in mean proportions of marker-positive cells. Categorical associations between immune checkpoint markers (e.g., OX40L^+^ vs. LAG3^+^ Tregs) were evaluated using χ² tests on tumor-level frequency data. Correlations between continuous variables were analyzed using Pearson’s correlation coefficient. Recurrence-free survival was assessed by Kaplan–Meier analysis with log-rank testing; cases without recurrence were censored at last follow-up. Patients were dichotomized using the median OX40L^+^ myeloid cell proportion, a standard non-parametric cutoff commonly applied in Kaplan–Meier analyses of small cohorts. Exploratory subgroup analyses, including stratification by patient sex, were performed but did not reveal significant associations. Analyses were performed using excel or Statistics Kingdom (http://www.statskingdom.com), and *p* < 0.05 was considered statistically significant.

## Results

Tumor samples were obtained from 30 patients with cutaneous primary melanoma, representing a range of clinicopathological characteristics, including variable vertical depths (1–15 mm), differing lymphocyte infiltration scores, and prognostic features such as ulceration, regional lymph node involvement, and recurrence (**Supplementary Table S1**).

### OX40L is broadly distributed across melanoma and adjacent skin compartments

**Figure 1:**
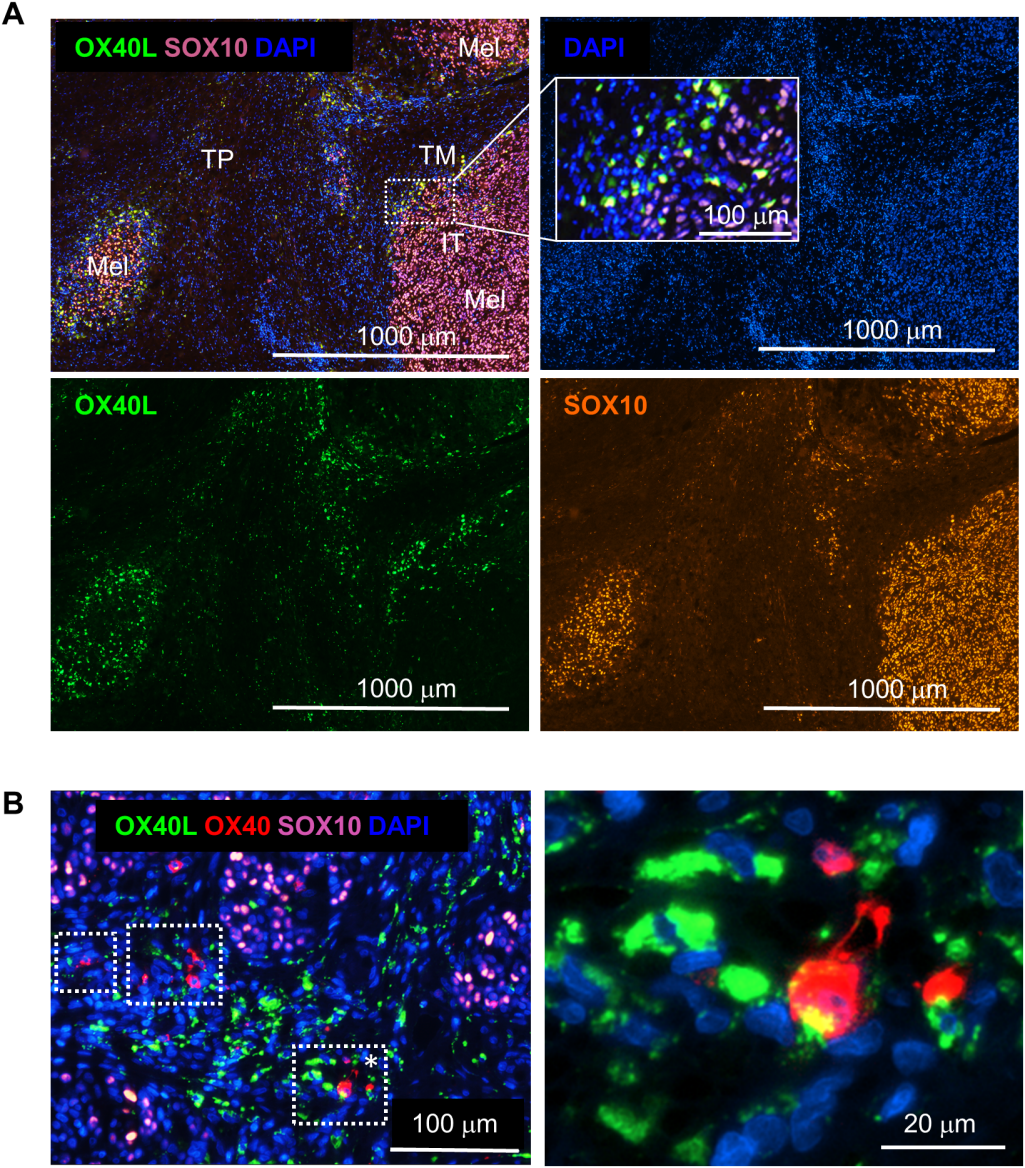
Spatial distribution of OX40L^+^ cells in the melanoma TME. **(a)** Representative mIF image showing OX40L (green), SOX10 (melanoma nuclei; orange when alone, magenta when overlaid with DAPI), and DAPI (blue). OX40L^+^ cells localize within intratumoral (IT), tumor–stroma margin (TM), and tumor-peripheral (TP) regions. **(b)** Representative image including OX40 (red), illustrating OX40L^+^ and OX40^+^ cells in spatial proximity suggestive of receptor–ligand co-localization (white-dashed squares). Right: higher-magnification view of the star-marked area.

To assess the abundance and spatial distribution of OX40L protein, we performed multiplex immunofluorescence (mIF) microscopy using antibodies against OX40L, OX40, the melanoma marker SOX10, and DAPI. OX40L^+^ cells were detected in intratumoral regions, the tumor–stroma interface, and peripheral tumor zones (**Figure 1a**). In some specimens, elongated OX40L^+^ cells arranged in linear or branched chains were observed among stromal cells, though the significance of this finding remains unclear (**Supplementary Figure S1a**).

Outside the tumor bed, OX40L^+^ cells were present in benign tissues including vascular endothelium, smooth muscle, myoepithelial cells of sweat glands, and epidermal or follicular keratinocytes (**Supplementary Figure S1b–d**), with similar patterns in adjacent normal skin (**Supplementary Figure S1c**). Notably, OX40^+^ cells were frequently located near OX40L^+^ cells within both tumors (**Figure 1b**) and normal skin structures (**Supplementary Figure S1e**), consistent with potential receptor–ligand interactions.

### OX40L^+^ cells vary widely in abundance among tumors, exceed OX40^+^ levels, and frequently colocalize with OX40^+^ counterparts in the melanoma TME

**Figure 2:**
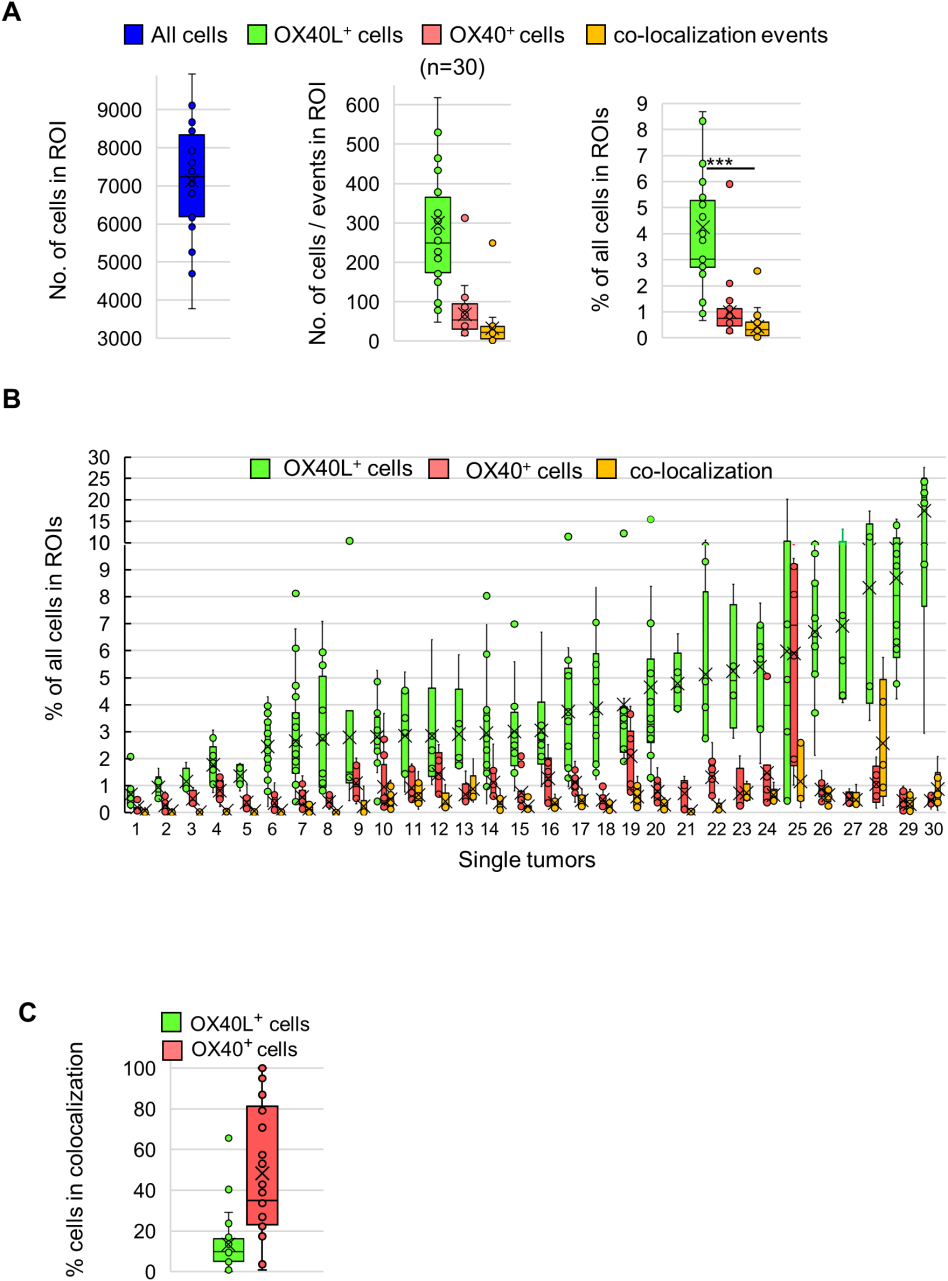
Quantitative analysis of OX40L^+^ and OX40^+^ cells and their spatial co-localization in melanoma. Multiplex immunofluorescence analysis of OX40L (green), OX40 (red), and DAPI (blue) was performed across 30 melanoma tumors. Quantification was based on 5–10 regions of interest (ROIs; 3 mm² each) per tumor. Co-localization was defined as a distance of <20 μm between OX40^+^ and OX40L^+^ cells. **(a)** Total nucleated cell counts (left), OX40L^+^ and OX40^+^ subpopulation counts (middle), and the corresponding percentages of each subpopulation relative to total cell counts (right) across tumors. Each dot represents the mean value across all ROIs within a single tumor. **(b)** Percentages of OX40L^+^ cells (green), OX40^+^ cells (red), and the number of co-localization events normalized to all DAPI^+^ nuclei for individual tumors, ordered by increasing OX40L^+^ frequency. Each dot represents a single ROI. **(c)** Box plots showing the proportion of OX40L^+^ cells (green) and OX40^+^ cells (red) within their respective populations that co-localize with the corresponding counterpart. The mean is indicated by a “X” mark and the median by a horizontal line. ***p < 0.001, Student’s t-test.

We next quantified OX40L^+^ and OX40^+^ cells and their proximity in the entire cohort of 30 tumors. Entire mIF-stained sections were manually screened to select 5–10 ROIs (3 mm² each) enriched for OX40L^+^ cells, which were analyzed with GEN5-prime software. Co-localization was defined as a distance of less than 20 μm between OX40^+^ and OX40L^+^ cells (**Supplementary Figure S2**), excluding skin structures. Across all ROIs, the mean nucleated cell density was 7,141 ± 1,371 cells per ROI (**Figure 2a**). The average number of OX40L^+^ cells per ROI across all tumors was higher than that of OX40^+^ cells (287 ± 197 vs. 67 ± 56; n = 30), corresponding to mean frequencies of 4.3 ± 3.2% and 1.0 ± 1.0% of all cells per ROI, respectively (**Figure 2a**). The abundance of OX40L^+^ cells varied widely among tumors, ranging from 1.0% to 17.5% of all nucleated cells, and exceeded that of OX40^+^ cells in most cases (**Figure 2b**). The mean number of co-localization events per ROI was 31.0 ± 44.0 (**Figure 2A**) and involved 48.0 ± 32.0% of OX40^+^ cells compared with 13.0 ± 13.0% of OX40L^+^ cells (**Figure 2c**). These findings indicate that OX40L^+^ cells are considerably more abundant than OX40^+^ cells, yet a substantial fraction of OX40^+^ cells are in close proximity with OX40L, pointing to potentially functional interactions within the melanoma TME.

### Infrequent expression of OX40L on melanoma cancer cells

**Figure 3:**
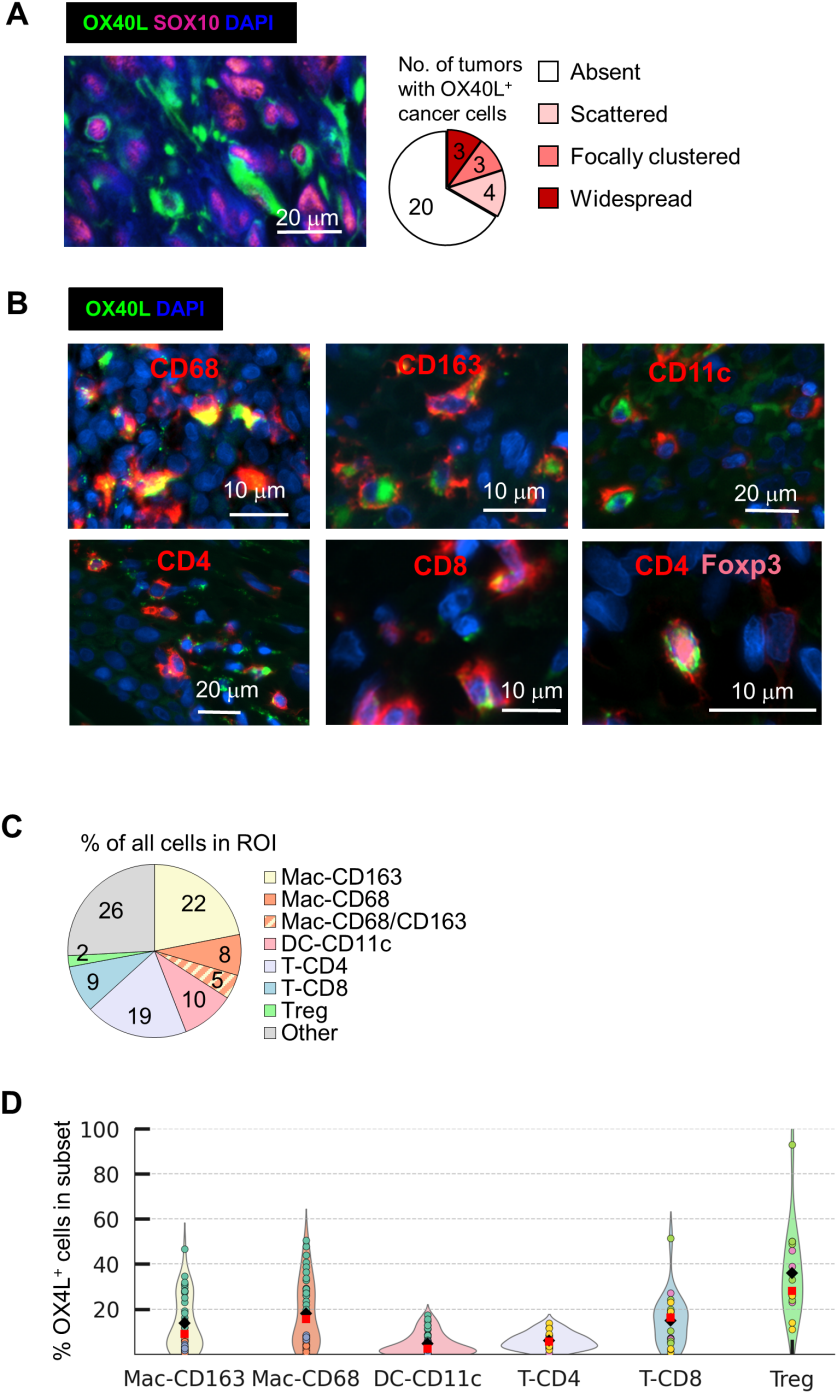
Cellular phenotyping of OX40L^+^ cells in melanoma tumors. **(a)** OX40L expression on SOX10^+^ melanoma cells was manually assessed in 30 tumors (5–10 ROIs per tumor; 3 mm² per ROI). Representative images of membrane-associated OX40L expression and a summary of prevalence across tumors are shown. Rare OX40L^+^ melanoma cells (<10 cells per ROI) were classified as “scattered” or “focally clustered,” whereas “widespread” distribution was defined as OX40L expression in ~10% of SOX10^+^ melanoma cells per ROI. **(b)** Exploratory OX40L immune cell phenotyping was performed on serial tumor sections from three melanoma cases. Representative multiplex immunofluorescence images illustrate OX40L expression in macrophages (CD68^+^, CD163^+^), dendritic cells (CD11c^+^), CD4^+^ T cells, CD8^+^ T cells, and Foxp3^+^ regulatory T cells. **(c)** Proportions of immune cell subsets within the TME across three tumors (8–15 ROIs per tumor). **(d)** Violin plots depicting the proportion of OX40L^+^ cells within each immune subset. Mac, macrophages; DC, dendritic cells; T, T cells. Each dot represents one ROI, dot colors represent tumor sample, and mean and median values are shown in red squares and black diamonds.

To assess OX40L expression in melanoma cells, we analyzed the entire tumor cohort (n = 30; 5–10 ROIs per tumor) for OX40L signal within <2 µm of SOX10^+^ nuclei. In 20 of 30 tumors, no OX40L expression was detected in melanoma cells. In seven tumors, only rare OX40L^+^/SOX10^+^ cells were identified across all ROIs (<10 cells per tumor), displaying either scattered or clustered distributions. In contrast, substantial and widespread OX40L expression was observed in only three of 30 tumors, with approximately 10% of SOX10^+^ melanoma cells expressing OX40L. Overall, melanoma cell–associated OX40L expression was detected in 10 of 30 tumors (33%), with patterns ranging from sparse to widespread (**Figure 3a**; **Supplementary Figure S3a–b**).

### Exploratory mIF analysis identifies OX40L expression across multiple immune cell types

To delineate the immune cell populations expressing OX40L within the melanoma tumor microenvironment, we performed an exploratory multiplex immunofluorescence analysis with software-based quantitative assessment on serial tissue sections from three melanoma tumors with sufficient material, using an expanded immune marker panel. OX40L expression was detected across multiple immune populations, including macrophages (CD68^+^: 18.0 ± 14.6%; CD163^+^: 13.7 ± 9.0%), dendritic cells (CD11c^+^: 4.0 ± 3.5%), CD4^+^ T cells (6.0 ± 3.7%), and CD8^+^ T cells (15.0 ± 11.0%) (**Figure 3b–d**; **Supplementary Figure S3c–g**). Rare OX40L expression was observed on CD19^+^ or CD20^+^ B cells, predominantly within tertiary lymphoid structures, but was not further analyzed due to its low frequency in this panel (**Supplementary Figure S3h**). Notably, OX40L expression was detected in CD4^+^Foxp3^+^ regulatory T cells (Tregs) (**Figure 3b**; **Supplementary Figure S3i**), with a mean prevalence of 35 ± 19% (**Figure 3d**). To our knowledge, OX40L expression in Tregs has not been previously reported, in contrast to the well-established expression of OX40 in this population (4, 8, 9). This finding prompted a subsequent cohort-wide analysis of OX40L expression in regulatory T cells.

### Tregs in the melanoma microenvironment express OX40L more frequently than OX40

**Figure 4:**
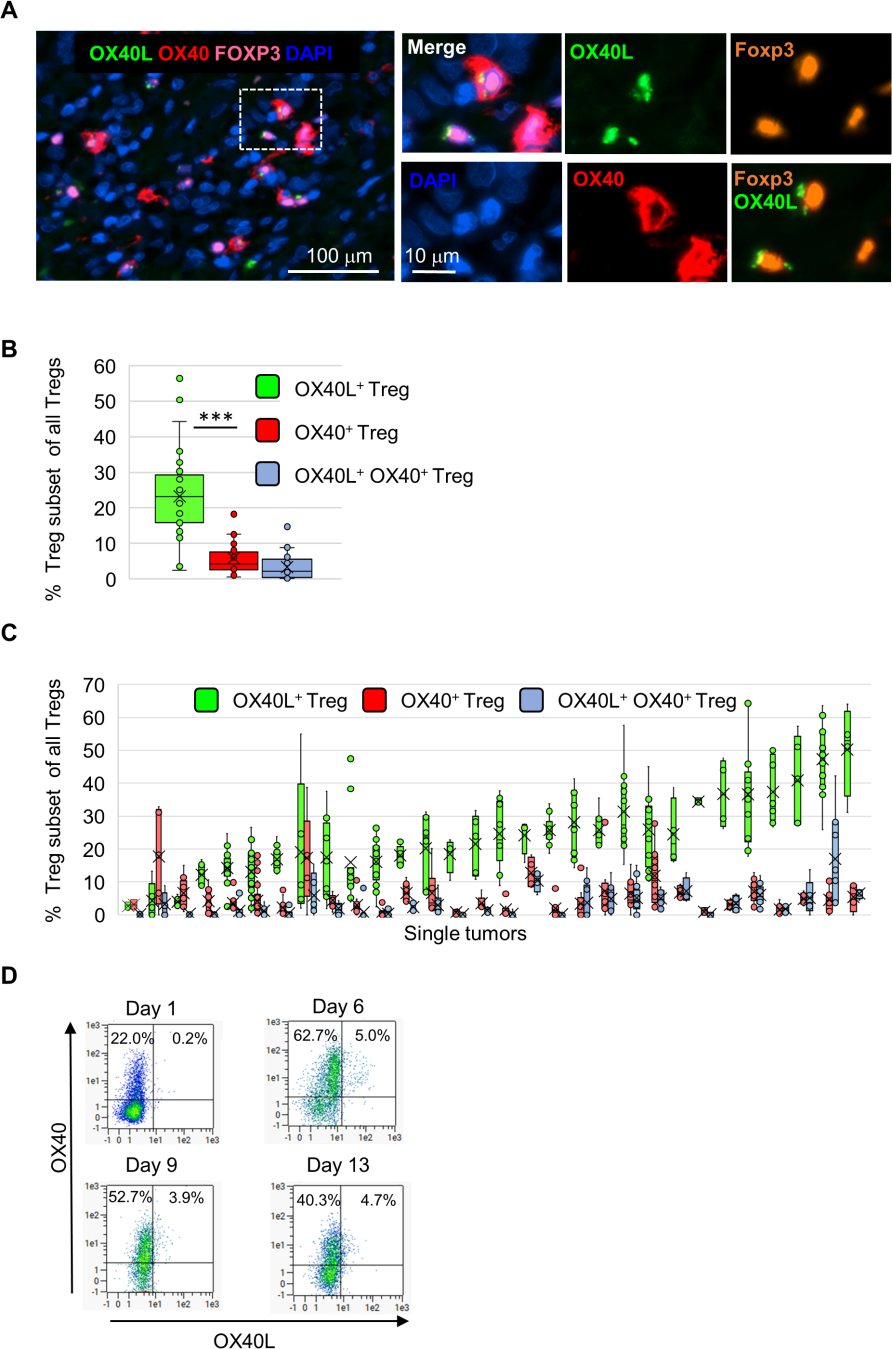
Expression of OX40L and OX40 on regulatory T cells in melanoma tumors and peripheral blood of a healthy donor. OX40L and OX40 expression were assessed on Foxp3^+^ regulatory T cells (Tregs) across 30 melanoma tumors using quantitative multiplex immunofluorescence analysis (10–20 ROIs per tumor; 3 mm² per ROI). **(a)** Representative images showing Foxp3^+^ Tregs within a melanoma tumor (Foxp3^+^ nuclei shown in orange when alone or magenta when overlaid with DAPI) co-expressing OX40L (green), OX40 (red), or both. Right panels show higher-magnification views of circled cells with individual color channels; all panels share the same scale bar, indicated in the DAPI image. **(b)** Box plots depicting the proportions of OX40L^+^, OX40^+^, and double-positive Tregs across 30 tumors. The mean is indicated by a “X” mark and the median by a horizontal line (***p < 0.001, Student’s t-test). Jittered dots represent the mean value per tumor across ROIs. **(c)** Box plots showing the prevalence of OX40L^+^ (green), OX40^+^ (red), and OX40L^+^/OX40L^+^ double-positive (blue) Tregs per tumor, ordered by OX40L^+^ frequency. Jittered dots represent individual ROIs. **(d)** Tregs isolated from peripheral blood of a healthy donor were enriched and expanded *in vitro* under IL-2/CD3/CD28 stimulation. The proportions of OX40L^+^ and OX40^+^ cells within live, singlet Foxp3^+^ regulatory T cells, as defined in the Methods, are shown over 13 days of culture.

Subsequent analyses of OX40L and OX40 expression were conducted across the entire cohort of 30 tumors. Using Foxp3 as a Treg marker, we identified distinct subsets defined by expression of OX40, OX40L, both, or neither (**Figure 4a**, **Supplementary Figure S4a–b**). Quantitative analysis (10–20 ROIs/tumor) showed significantly higher frequencies of OX40L^+^ compared with OX40^+^ Tregs (23.0 ± 12.5% vs. 6.0 ± 4.6%, p < 0.001), with 3.5% co-expressing both (**Figure 4b**). The prevalence of OX40L^+^ Tregs varied widely among individual tumors (2.5–56%) and in most cases exceeded that of OX40^+^ Tregs (0.6–18.5%), with no correlation between the two subsets (Pearson r = −0.165) (**Figure 4c**).

To corroborate that Treg are capable of expressing OX40L protein, Tregs enriched from the peripheral blood of a healthy donor were expanded under stimulatory culture conditions. Serial flow cytometry throughout 13 days in culture confirmed that the majority of Foxp3^+^ cells (88–98%) co-expressed CD4 and CD25 (**Supplementary Figure S4d**), validating their identity as Tregs. Within this population, a small fraction expressed OX40L (0.2–5.0%), whereas OX40 expression was consistently much higher (22–63%) (**Figure 4d**). Gene expression analysis of total cells on days 9 and 13 further confirmed lower OX40L mRNA compared with OX40, IL-2RA, and Foxp3 (**Supplementary Figure S4e**). These findings clearly confirm that Tregs can express OX40L mRNA and protein, although its expression in blood-derived Tregs was rare compared with predominant OX40 expression, suggesting tissue-specific and context-specific regulation.

### Differential co-expression of OX40L and OX40 with other immune checkpoint proteins in tumor-resident Tregs

**Figure 5:**
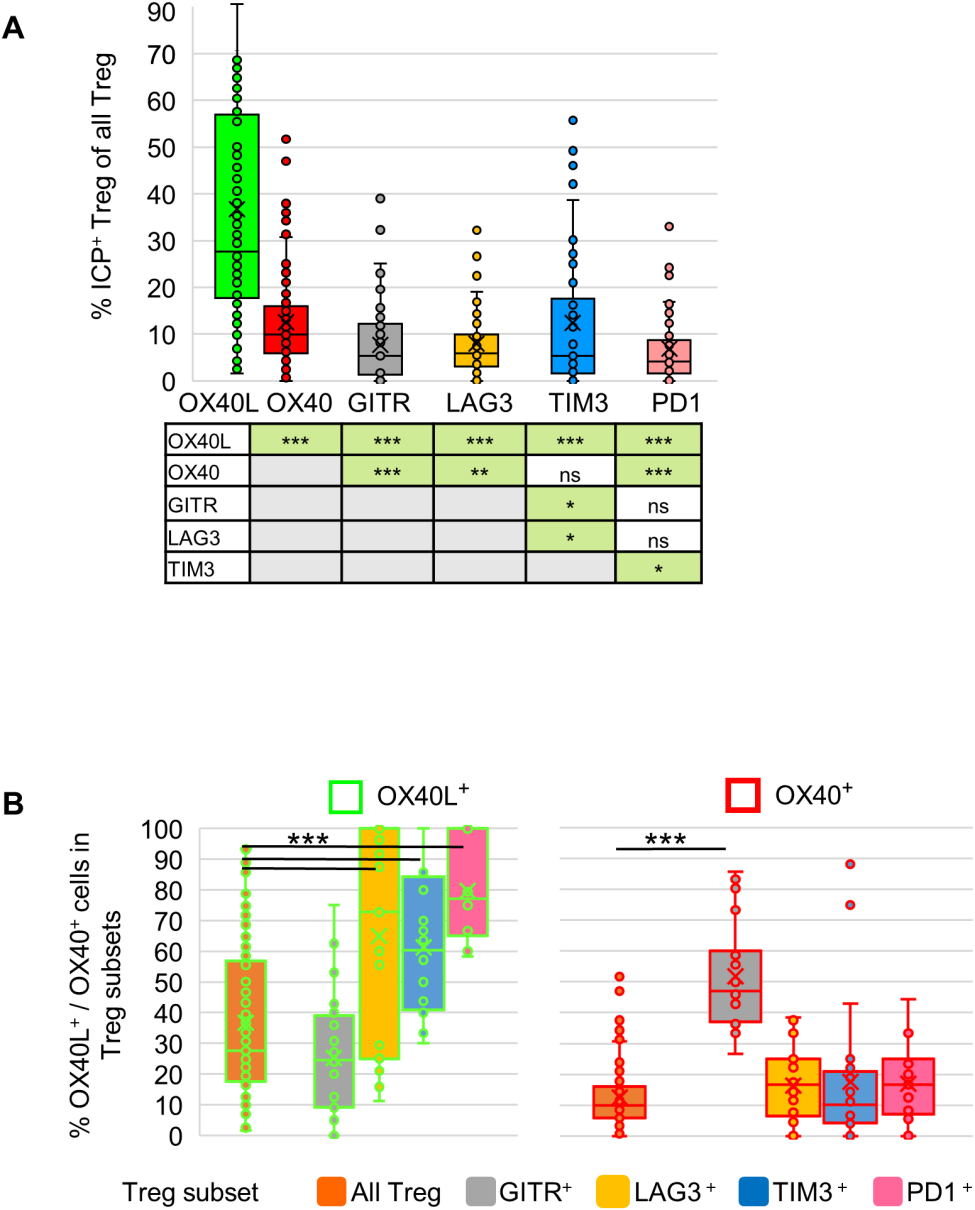
Co-expression of OX40 and OX40L with other immune checkpoint proteins (ICPs) in tumor-resident Tregs. Exploratory quantitative mIF analysis of three melanoma tumors (8–15 ROIs per tumor). **(a)** Box plots showing the proportions of Tregs expressing each ICP (OX40L, OX40, GITR, LAG3, TIM3, and PD1) among all Tregs. Each dot represents one ROI. Statistical comparisons between all groups are summarized in the accompanying table below. **(b)** Box plots showing the proportions of OX40L^+^ and OX40^+^ cells within All Tregs, and within GITR^+^, LAG3^+^, TIM3^+^, and PD1^+^ Treg subsets. Each dot represents one ROI. Statistical significance p-values are shown *p<0.05, **p<0.01, ***p<0.001.

We next examined the co-expression of OX40 and OX40L with additional immune checkpoint proteins (ICPs) commonly associated with activated Tregs, including LAG3, TIM3, PD1 and GITR (representative images in **Supplementary Figure S5a–b**). An exploratory quantitative analysis of three tumors (8–15 ROIs per tumor) revealed that among all Tregs, OX40L expression was not only more frequent than OX40 (37% vs. 13%, consistent with **Figure 4b**) but also exceeded that of other ICPs, including LAG3 (8%), TIM3 (12%), PD1 (7%) and GITR (8%) (**Figure 5a**).

Within ICP^+^ Treg subsets, OX40L expression was significantly higher than expected in LAG3^+^, TIM3^+^, and PD1^+^ cells (65%, 61%, and 80%, respectively) compared with its frequency in the total Treg population (37%) (**Figure 5b**, left; χ² test, p < 0.05). In contrast, OX40 expression was significantly higher than expected within the GITR^+^ subset (52% vs. 13%; χ² test, p < 0.05), but showed no association with LAG3^+^, TIM3^+^, or PD1^+^ subsets (16%, 17%, and 17%, respectively) (**Figure 5b**, right). These findings indicate that OX40L expression in Tregs is not only more frequent than other ICPs but also selectively associated with LAG3, TIM3, and PD1 expression, whereas OX40 is preferentially linked to GITR^+^ Tregs. This pattern raises the possibility that OX40 and OX40L serve distinct roles within the Treg compartment, warranting further investigation to clarify their functional relevance in Treg regulation.

### Exploratory association between myeloid OX40L expression and disease recurrence

**Figure 6:**
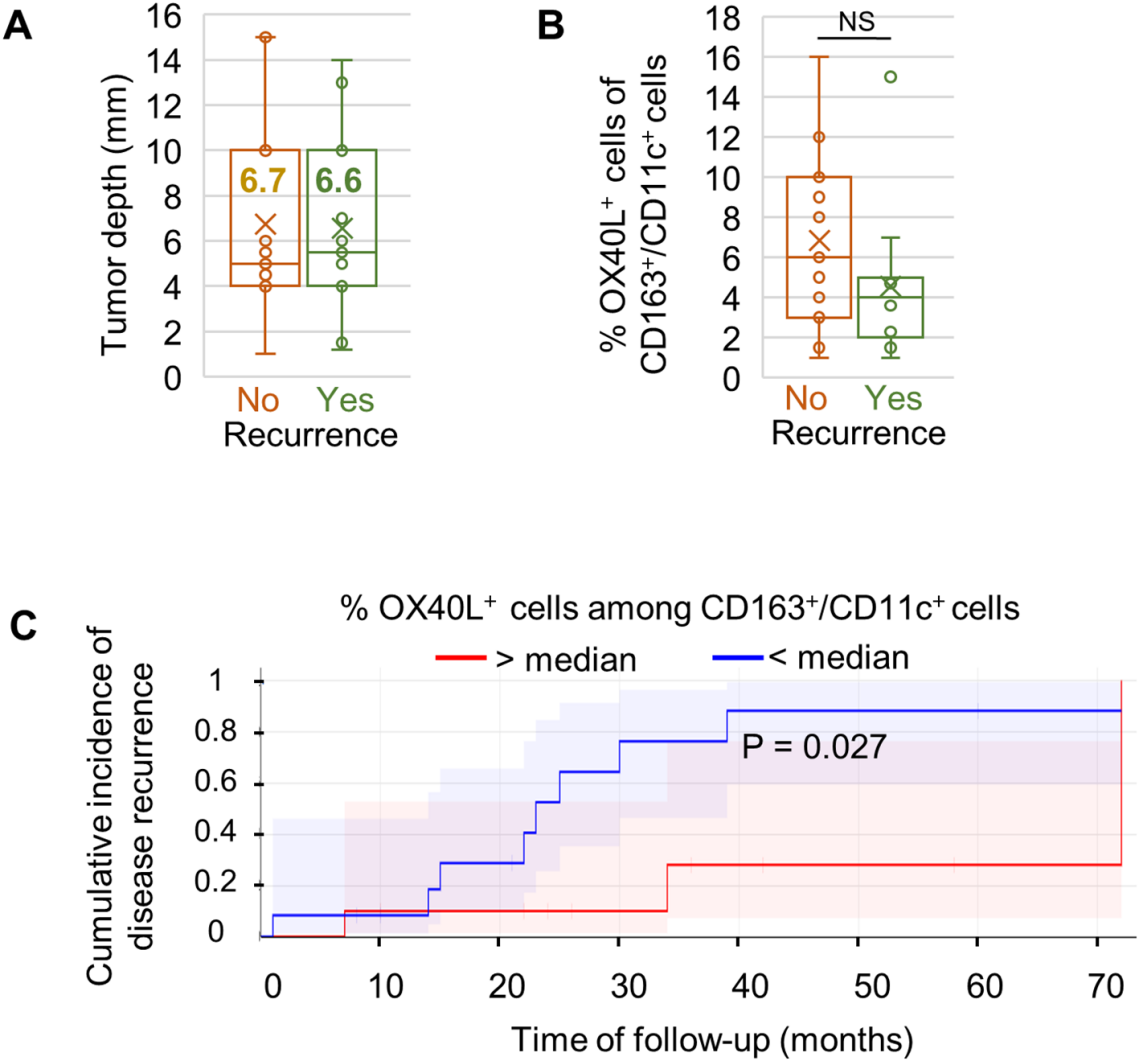
Association of myeloid OX40L expression with melanoma recurrence. **(a)** Tumor thickness matched between recurrent (n = 11) and non-recurrent (n = 11) patients. **(b)** Box plots show proportions of OX40L^+^ cells within the CD163^+^/CD11c^+^ myeloid compartment. **(c)** Kaplan–Meier curves of cumulative recurrence incidence in patients stratified by OX40L^+^ myeloid-cell proportions below versus above the cohort median (non-optimized cutoff); shaded areas indicate 95% confidence intervals.

Finally, we explored associations between OX40L expression and clinical outcome. Cases with disease recurrence (n = 11) were matched to non-recurrent cases (n = 11) by tumor thickness to control for this major prognostic factor (**Figure 6a** and **Supplementary Figure S6a**). No differences were observed between the two groups in the overall abundance of OX40L^+^ or OX40^+^ cells, their spatial co-localization, or the prevalence of Treg subsets.

Within the CD163^+^/CD11c^+^ myeloid compartment (representative images in **Supplementary Figure S6b**), the proportion of OX40L^+^ cells was higher in non-recurrent patients (6.9 ± 4.6%) compared with recurrent patients (4.5 ± 3.7%) (**Figure 6b**; **Supplementary Figure S6a**). When patients were stratified by the median proportion of OX40L^+^ myeloid cells (4.7%), Kaplan–Meier analysis showed a difference in cumulative recurrence incidence between groups (p = 0.027; **Figure 6c**), with higher myeloid OX40L expression observed in patients without recurrence.

## Discussion

Our study provides novel evidence of OX40L protein expression in the microenvironment of primary melanoma, demonstrating marked variability across tumors and involvement of multiple cell types. Overall, OX40L^+^ cells were more abundant than OX40^+^ cells, and OX40^+^ cells frequently localized near OX40L^+^ counterparts, suggesting potential receptor–ligand interactions. Similarly, the co-localization of PD1 and PDL1 on neighboring cells in melanoma has been interpreted as evidence of functional engagement in cancer immunoediting (10), implying that OX40–OX40L proximity may carry comparable functional significance. In line with other reports (11–13), OX40L^+^ cells were commonly found in melanoma-adjacent and normal skin structures, nearby OX40^+^ lymphocytes, indicative of potential interactions outside the tumor bed as well (14, 15).

A few earlier studies have reported OX40L protein expression in lung, ovarian, and pancreatic cancers, spanning both tumor and immune compartments (16–19). In pancreatic cancer, higher frequencies of OX40L on tumor cells or macrophages correlated with improved survival in a cohort of 255 patients (16). We identified OX40L^+^ cancer cells in one-third of melanoma tumors, at variable and often sparse densities, but found no association with clinical outcome in our small cohort. In comparison, OX40L protein was consistently detected on myeloid cells—including macrophages and dendritic cells—across all melanoma tumors examined. This observation aligns with murine studies (20–22), where OX40L expression on myeloid cells was shown to promote antitumor immunity. Notably, in a tumor depth–matched melanoma sub-cohort (n = 22), higher proportions of OX40L^+^ myeloid cells (CD163^+^/CD11c^+^) were observed in patients without disease recurrence. While this exploratory association reached statistical significance in this limited cohort, its clinical significance remains to be clarified in larger, independent studies.

Accordingly, preclinical mouse models have demonstrated that ectopic overexpression of OX40L in cancer cells or other cell types at the tumor site can effectively stimulate antitumor immunity (23–26). Building on these findings, early-phase clinical trials have tested intratumoral delivery of OX40L mRNA, or combined OX40L/IL-23/IL-36γ mRNA, with or without PD-1 blockade. These interventions increased T-cell abundance and proliferation, alongside upregulation of immune gene signatures linked to antitumor responses (27–30). Importantly, our results indicate a relatively low prevalence of OX40, which may restrict its capacity to interact with OX40L. This observation may help guide future efforts to identify patient groups who could benefit from OX40L-overexpression strategies.

Within the lymphocytic lineage, both CD4^+^ and CD8^+^ T cells expressed OX40L protein—a notable finding, as T cells are generally defined by OX40 expression (4) and were only rarely reported to express OX40L (31, 32). Strikingly, OX40L was more frequent than OX40 in intratumoral Tregs, whereas validation in blood-derived Tregs revealed the opposite pattern, with OX40 protein predominating.

A critical mechanistic question is whether OX40L detected on tumor-infiltrating Tregs reflects trogocytosis or endogenous ligand expression. Trogocytosis in Tregs is a well-established mechanism in which CTLA-4 expressed on Tregs mediates the removal of CD80/CD86 from antigen-presenting cells, thereby enhancing immunosuppression through loss of APC-derived costimulatory signals required for effector T-cell activation and increased availability of PD-L1 to engage PD-1 on T cells (33, 34). Similarly, OX40L expression on Tregs could plausibly arise from membrane acquisition from neighboring OX40L^+^ APCs within the tumor microenvironment. However, we additionally demonstrate that peripheral blood Tregs retain the capacity to induce endogenous OX40L expression under defined stimulatory conditions, indicating intrinsic expression potential. Notably, only limited precedent exists for ligand–receptor co-expression in T cells, with one example being the 4-1BB/4-1BBL axis, where T-cell–expressed 4-1BBL has been proposed to deliver counter-regulatory inhibitory signals (35). Further studies distinguishing trogocytosis from endogenous ligand expression are essential for interpreting the role of OX40-OX40L in Treg within the TME.

Furthermore, intratumoral OX40^+^ Tregs displayed a significantly different co-expression profile of immune checkpoint proteins (ICPs) than OX40L^+^ Tregs, indirectly suggesting a different activation state in each of these subgroups. Clearly, functional assays with live cells are needed to characterize the activation state of the OX40L^+^ or OX40^+^ Treg. Taken together, our findings raise the hypothesis that OX40L^+^ Tregs may participate in local OX40-dependent immune interactions within the melanoma tumor microenvironment. The functional relevance of these interactions cannot be inferred from the present data and will require future mechanistic studies—such as *in vitro* Treg suppression assays or OX40L-blocking experiments—to determine whether OX40L expression influences Treg activity or survival. This hypothesis is conceptually supported by prior experimental studies by others, showing that OX40 pathway modulation, including treatment with OX40 agonist antibodies, can suppress Treg function and promote tumor rejection (6, 36–38).

In our cohort, no significant associations were detected between OX40^+^ or OX40L^+^ Tregs and clinical outcomes, possibly reflecting the limited sample size. Further investigation into potential associations with additional parameters, such as response to immunotherapy, is warranted—particularly given prior reports linking enhanced Treg suppressive activity with reduced efficacy of ICI therapy (39–41).

## Conclusion

In summary, our findings suggest a multifaceted role for OX40L in the melanoma microenvironment, potentially shaping interactions among tumor, immune, and skin compartments. Its diverse expression patterns, together with their prognostic and functional implications, encourage further investigation into its potential as a biomarker and therapeutic target.

## Data Availability

The original contributions presented in the study are included in the article/**Supplementary Material**. Further inquiries can be directed to the corresponding author/s.

## References

[1] Korman AJ Garrett-Thomson SC Lonberg N . The foundations of immune checkpoint blockade and the ipilimumab approval decennial. Nat Rev Drug Discov. (2022) 21:509–28. doi: 10.1038/s41573-021-00345-8, PMID: 34937915

[2] Emens LA Romero PJ Anderson AC Bruno TC Capitini CM Collyar D . Challenges and opportunities in cancer immunotherapy: a Society for Immunotherapy of Cancer (SITC) strategic vision. J Immunother Cancer. (2024) 12:e009063. doi: 10.1136/jitc-2024-009063, PMID: 38901879 PMC11191773

[3] Croft M So T Duan W Soroosh P . The significance of OX40 and OX40L to T-cell biology and immune disease. Immunol Rev. (2009) 229:173–91. doi: 10.1111/j.1600-065X.2009.00766.x, PMID: 19426222 PMC2729757

[4] Croft M . Control of immunity by the TNFR-related molecule OX40 (CD134). Annu Rev Immunol. (2010) 28:57–78. doi: 10.1146/annurev-immunol-030409-101243, PMID: 20307208 PMC2882161

[5] Edner NM Carlesso G Rush JS Walker LSK . Targeting co-stimulatory molecules in autoimmune disease. Nat Rev Drug Discov. (2020) 19:860–83. doi: 10.1038/s41573-020-0081-9, PMID: 32939077

[6] Thapa B Kato S Nishizaki D Miyashita H Lee S Nesline MK . OX40/OX40 ligand and its role in precision immune oncology. Cancer Metastasis Rev. (2024) 43:1001–13. doi: 10.1007/s10555-024-10184-9, PMID: 38526805 PMC11300540

[7] Roszik J Markovits E Dobosz P Layani A Slabodnik-Kaner K Baruch EN . TNFSF4 (OX40L) expression and survival in locally advanced and metastatic melanoma. Cancer Immunol Immunother. (2019) 68:1493–500. doi: 10.1007/s00262-019-02382-0, PMID: 31501955 PMC11028300

[8] Piconese S Valzasina B Colombo MP . OX40 triggering blocks suppression by regulatory T cells and facilitates tumor rejection. J Exp Med. (2008) 205:825–39. doi: 10.1084/jem.20071341, PMID: 18362171 PMC2292222

[9] Willoughby J Griffiths J Tews I Cragg MS . OX40: Structure and function - What questions remain? Mol Immunol. (2017) 83:13–22. doi: 10.1016/j.molimm.2017.01.006, PMID: 28092803

[10] Nirmal AJ Maliga Z Vallius T Quattrochi B Chen AA Jacobson CA . The spatial landscape of progression and immunoediting in primary melanoma at single-cell resolution. Cancer Discov. (2022) 12:1518–41. doi: 10.1158/2159-8290.CD-21-1357, PMID: 35404441 PMC9167783

[11] Burgess JK Carlin S Pack RA Arndt GM Au WW Johnson PRA . Detection and characterization of OX40 ligand expression in human airway smooth muscle cells: a possible role in asthma? J Allergy Clin Immunol. (2004) 113:683–9. doi: 10.1016/j.jaci.2003.12.311, PMID: 15100674

[12] Imura A Hori T Imada K Ishikawa T Tanaka Y Maeda M . The human OX40/gp34 system directly mediates adhesion of activated T cells to vascular endothelial cells. J Exp Med. (1996) 183:2185–95. doi: 10.1084/jem.183.5.2185, PMID: 8642328 PMC2192546

[13] Ilves T Harvima IT . OX40 ligand and OX40 are increased in atopic dermatitis lesions but do not correlate with clinical severity. J Eur Acad Dermatol Venereol. (2013) 27:e197–205. doi: 10.1111/j.1468-3083.2012.04587.x, PMID: 22646697

[14] Mestas J Crampton SP Hori T Hughes CCW . Endothelial cell co-stimulation through OX40 augments and prolongs T cell cytokine synthesis by stabilization of cytokine mRNA. Int Immunol. (2005) 17:737–47. doi: 10.1093/intimm/dxh255, PMID: 15908450

[15] Matsumura Y Imura A Hori T Uchiyama T Imamura S . Localization of OX40/gp34 in inflammatory skin diseases: a clue to elucidate the interaction between activated T cells and endothelial cells in infiltration. Arch Dermatol Res. (1997) 289:653–6. doi: 10.1007/s004030050255, PMID: 9444389

[16] Chen X Ma H Mo S Zhang Y Lu Z Yu S . Analysis of the OX40/OX40L immunoregulatory axis combined with alternative immune checkpoint molecules in pancreatic ductal adenocarcinoma. Front Immunol. (2022) 13:942154. doi: 10.3389/fimmu.2022.942154, PMID: 35936015 PMC9352865

[17] Chen P Wang H Zhao L Guo H Zhang L Zhang W . Immune checkpoints OX40 and OX40L in small-cell lung cancer: predict prognosis and modulate immune microenvironment. Front Oncol. (2021) 11:713853. doi: 10.3389/fonc.2021.713853, PMID: 34900670 PMC8652148

[18] Porciuncula A Morgado M Gupta R Syrigos K Meehan R Zacharek SJ . Spatial mapping and immunomodulatory role of the OX40/OX40L pathway in human non-small cell lung cancer. Clin Cancer Res An Off J Am Assoc Cancer Res. (2021) 27:6174–83. doi: 10.1158/1078-0432.CCR-21-0987, PMID: 34518312 PMC8595671

[19] Sandström Gerdtsson A Knulst M Botling J Mezheyeuski A Micke P Ek S . Phenotypic characterization of spatial immune infiltration niches in non-small cell lung cancer. Oncoimmunology. (2023) 12:2206725. doi: 10.1080/2162402X.2023.2206725, PMID: 37139184 PMC10150622

[20] Jenkins SJ Perona-Wright G Worsley AGF Ishii N MacDonald AS . Dendritic cell expression of OX40 ligand acts as a costimulatory, not polarizing, signal for optimal Th2 priming and memory induction in *vivo*. J Immunol. (2007) 179:3515–23. doi: 10.4049/jimmunol.179.6.3515, PMID: 17785785

[21] Gajdasik DW Gaspal F Halford EE Fiancette R Dutton EE Willis C . Th1 responses *in vivo* require cell-specific provision of OX40L dictated by environmental cues. Nat Commun. (2020) 11:3421. doi: 10.1038/s41467-020-17293-3, PMID: 32647184 PMC7347572

[22] Lee CYC Kennedy BC Richoz N Dean I Tuong ZK Gaspal F . Tumour-retained activated CCR7(+) dendritic cells are heterogeneous and regulate local anti-tumour cytolytic activity. Nat Commun. (2024) 15:682. doi: 10.1038/s41467-024-44787-1, PMID: 38267413 PMC10808534

[23] Shibahara I Saito R Zhang R Chonan M Shoji T Kanamori M . OX40 ligand expressed in glioblastoma modulates adaptive immunity depending on the microenvironment: a clue for successful immunotherapy. Mol Cancer. (2015) 14:41. doi: 10.1186/s12943-015-0307-3, PMID: 25744203 PMC4339477

[24] Zaini J Andarini S Tahara M Saijo Y Ishii N Kawakami K . OX40 ligand expressed by DCs costimulates NKT and CD4+ Th cell antitumor immunity in mice. J Clin Invest. (2007) 117:3330–8. doi: 10.1172/JCI32693, PMID: 17975668 PMC2045612

[25] Liu S Li F Ma Q Du M Wang H Zhu Y . OX40L-armed oncolytic virus boosts T-cell response and remodels tumor microenvironment for pancreatic cancer treatment. Theranostics. (2023) 13:4016–29. doi: 10.7150/thno.83495, PMID: 37554264 PMC10405835

[26] Andarini S Kikuchi T Nukiwa M Pradono P Suzuki T Ohkouchi S . Adenovirus vector-mediated *in vivo* gene transfer of OX40 ligand to tumor cells enhances antitumor immunity of tumor-bearing hosts. Cancer Res. (2004) 64:3281–7. doi: 10.1158/0008-5472.can-03-3911, PMID: 15126371

[27] Patel M Jimeno A Wang D Stemmer S Bauer T Sweis R . 539 Phase 1 study of mRNA-2752, a lipid nanoparticle encapsulating mRNAs encoding human OX40L/IL-23/IL-36γ, for intratumoral (ITu) injection +/- durvalumab in advanced solid tumors and lymphoma. J Immunother Cancer. (2021) 9:Abstract 539. doi: 10.1136/jitc-2021-SITC2021.539

[28] Abadier M Sullivan RJ Chow J Sehgal V Sweis RF Daud A . Abstract 6552: Intratumoral (ITu) delivery of mRNA-2752 encoding human OX40L/IL-23/IL-36γ in combination with durvalumab induces an immunostimulatory effect within the tumor microenvironment (TME) of patients with advanced solid tumors. Cancer Res. (2024) 84:6552. doi: 10.1158/1538-7445.AM2024-6552

[29] Sullivan RJ Yeku OO Teoh D Gupta S Matei D Laino AS . First-in-human phase I/II, open-label study of mRNA-2416 alone or combined with durvalumab in patients with advanced solid tumors and ovarian cancer. Oncologist. (2025) 30:oyaf115. doi: 10.1093/oncolo/oyaf115, PMID: 40515479 PMC12166121

[30] Ramalingam K Woody R Glencer A Schwartz CJ Mori H Wong J . Intratumoral injection of mRNA-2752 and pembrolizumab for high-risk ductal carcinoma *in situ*: A phase 1 nonrandomized clinical trial. JAMA Oncol. (2025) 11:288–92. doi: 10.1001/jamaoncol.2024.5927, PMID: 39821301 PMC11926637

[31] Ishii N Takahashi T Soroosh P Sugamura K . OX40-OX40 ligand interaction in T-cell-mediated immunity and immunopathology. Adv Immunol. (2010) 105:63–98. doi: 10.1016/S0065-2776(10)05003-0, PMID: 20510730

[32] Soroosh P Ine S Sugamura K Ishii N . OX40-OX40 ligand interaction through T cell-T cell contact contributes to CD4 T cell longevity. J Immunol. (2006) 176:5975–87. doi: 10.4049/jimmunol.176.10.5975, PMID: 16670306

[33] Qureshi OS Zheng Y Nakamura K Attridge K Manzotti C Schmidt EM . Trans-endocytosis of CD80 and CD86: a molecular basis for the cell-extrinsic function of CTLA-4. Science. (2011) 332:600–3. doi: 10.1126/science.1202947, PMID: 21474713 PMC3198051

[34] Tekguc M Wing JB Osaki M Long J Sakaguchi S . Treg-expressed CTLA-4 depletes CD80/CD86 by trogocytosis, releasing free PD-L1 on antigen-presenting cells. Proc Natl Acad Sci U.S.A. (2021) 118:e2023739118. doi: 10.1073/pnas.2023739118, PMID: 34301886 PMC8325248

[35] Eun S-Y Lee S-W Xu Y Croft M . 4-1BB ligand signaling to T cells limits T cell activation. J Immunol. (2015) 194:134–41. doi: 10.4049/jimmunol.1401383, PMID: 25404362 PMC4272921

[36] So T Lee S-W Croft M . Immune regulation and control of regulatory T cells by OX40 and 4-1BB. Cytokine Growth Factor Rev. (2008) 19:253–62. doi: 10.1016/j.cytogfr.2008.04.003, PMID: 18508403 PMC2486494

[37] Marabelle A Kohrt H Sagiv-Barfi I Ajami B Axtell RC Zhou G . Depleting tumor-specific Tregs at a single site eradicates disseminated tumors. J Clin Invest. (2013) 123:2447–63. doi: 10.1172/JCI64859, PMID: 23728179 PMC3668834

[38] Bulliard Y Jolicoeur R Zhang J Dranoff G Wilson NS Brogdon JL . OX40 engagement depletes intratumoral Tregs via activating FcγRs, leading to antitumor efficacy. Immunol Cell Biol. (2014) 92:475–80. doi: 10.1038/icb.2014.26, PMID: 24732076

[39] Kamada T Togashi Y Tay C Ha D Sasaki A Nakamura Y . PD-1(+) regulatory T cells amplified by PD-1 blockade promote hyperprogression of cancer. Proc Natl Acad Sci U.S.A. (2019) 116:9999–10008. doi: 10.1073/pnas.1822001116, PMID: 31028147 PMC6525547

[40] Tay C Tanaka A Sakaguchi S . Tumor-infiltrating regulatory T cells as targets of cancer immunotherapy. Cancer Cell. (2023) 41:450–65. doi: 10.1016/j.ccell.2023.02.014, PMID: 36917950

[41] Tan CL Kuchroo JR Sage PT Liang D Francisco LM Buck J . PD-1 restraint of regulatory T cell suppressive activity is critical for immune tolerance. J Exp Med. (2021) 218:e20182232. doi: 10.1084/jem.20182232, PMID: 33045061 PMC7543091

