# Supplementary material for: Prognostic immunological implications of OX40L expression in the tumor microenvironment of melanoma
Source: Front Immunol. 2026 Jan 23;17:1745742. doi: 10.3389/fimmu.2026.1745742 (PMC12876231; doi:10.3389/fimmu.2026.1745742)
Supplement: Supplementary file 1 [file DataSheet1.pdf]

Supplemental Materials & Methods

Supplemental Tables

Table S1. Patient characteristics

No. of patients in study	30
Gender	
Male	19
Female	11
Age	
Mean (range), y	74(50-90)
Melanoma subtype	
Superficial spreading melanoma	8
Nodular melanoma	9
Not specified	13
Clark classification	
I	0
II	0
III	2
IV	20
V	6
Not specified	2
Site of origin (Primary)	
Trunk	8
Head	7
Upper limb	5
Lower limb	9
Not specified	1
Breslow depth	
Mean (range), mm	6(1-15)
Mitoses	
Mean (range)	7(1-40)
TILs (H&E IHC)	
Present, brisk	9
Present, non-brisk	16
Not specified	3

Ulceration	
Present	11
Absent	9
Not specified, not applicable	10
Lymph node metastasis	
Positive	5
Negative	10
Not submitted	15
Last known disease status	
Tumor free	14
Loco-regional recurrence	11
Data not available	5
Follow-up	
Mean (range), months	35 (8-72)
Data not available	2
Vital status	
Alive	19
Dead	9
Data not available	2

Table S2A

Primary antibodies for multiplex immunofluorescence analysis

Target	PROVIDER	CAT#	Antibody	Actual dilution
CD11c	CST	45581	CD11c (D3V1E) XP® Rabbit mAb	1:250
CD163	CST	93498	CD163 (D6U1J) Rabbit mAb	1:250
CD19	Cell Marque	119R-16	CD19 EP169 Rabbit mAB	1:50
CD20	Dako Agilent	M075501-2	CD20 L26 Mouse mAB	1:400
CD4	CST	48274	CD4 (EP204) Rabbit mAb	1:100
CD68	Zytomed	MSK055	KP1 Mouse mAB	1:100
CD8	CST	85336	CD8α (D8A8Y) Rabbit mAb	1:100
FoxP3	CST	98377	FoxP3 (D2W8E™) Rabbit mAb	1:100
GITR	CST	10419	GITR (D5V7P) Rabbit mAb	1:100
LAG3	CST	15372	LAG3 (D2G4O™) XP® Rabbit mAb	1:100
OX40	CST	98785	OX40 (ACT35) Mouse mAb	1:100
OX40L	CST	59036	X40L (D6K7R) Rabbit mAb (IHC Specific)	1:100
PD1	CST	86163	PD-1 (D4W2J) XP® Rabbit mAb	1:100
SOX10	CST	69661	Sox10 (E6B6I) XP® Rabbit mAb	1:200
SOX10	Cell Marque	383R-14	Sox -10 (EP268) Rabbit MAb	1:200
TIM3	CST	45208	TIM-3 (D5D5R™) XP® Rabbit mAb	1:100

Table S2B

Secondary antibodies and fluorophores used for multiplex immunofluorescence analysis

Fluorophore	Provider	CAT#	Channel (Ex, Em)
Alexa Fluor™ 647 Tyramide SuperBoost™ Kit, goat anti-mouse IgG	Invitrogen Thermo Fisher Scientific	B40916	CY5 (628, 685)
Alexa Fluor™ 647 Tyramide SuperBoost™ Kit, goat anti-rabbit IgG	Invitrogen Thermo Fisher Scientific	B40926	CY5 (628, 685)
Alexa Fluor™ 488 Tyramide SuperBoost™ Kit, goat anti-mouse IgG	Invitrogen Thermo Fisher Scientific	B40941	GFP (469, 525)
Alexa Fluor™ 488 Tyramide SuperBoost™ Kit, goat anti-rabbit IgG	Invitrogen Thermo Fisher Scientific	B40922	GFP (469, 525)
Alexa Fluor™ 555 Tyramide SuperBoost™ Kit, goat anti-mouse IgG	Invitrogen Thermo Fisher Scientific	B40913	RFP (531, 593)
Alexa Fluor™ 555 Tyramide SuperBoost™ Kit, goat anti-rabbit IgG	Invitrogen Thermo Fisher Scientific	B40923	RFP (531, 593)
EverBrite™ Hardset Mounting Medium with DAPI	Biotium	23004	DAPI (377, 447)

Table S3A Antibodies used for flow cytometry

Target	Ab clone	Ab Isotype	Vendor	Catalog #	Dilution
Cell viability			Miltenyi Biotec	130-130-403	1:100
CD4		Recombinant human IgG1	Miltenyi Biotec	130-113-792	1:50
CD25	REA570	Recombinant human IgG1	Miltenyi Biotec	130-123-585	1:25
OX40	ACT35	Mouse IgG1	Miltenyi Biotec	130-128-895	1:50
OX40L	159403	Mouse IgG1	R&D systems	Fab-105-41G	1:40
Foxp3	REA1253	Recombinant human IgG1	Miltenyi Biotec	130-125-587	1:50

Table S3B Ab-conjugated fluorophores used for flow cytometry

Target	Fluorophore	Laser	Filter
Cell viability		Violet405	450/50
CD4	VioGreen	Violet405	525/50
CD25	APC-Vio 770	Red640	750LP
OX40	APC	Red640	655-730
OX40L	AlexaFluor488	Blue488	525/50
Foxp3	PE	Blue488	585/40

Table S4 – Primer sequences for RT-PCR

Target Gene	RefSeq Accession No.	A strand	Oligo Sequence
OX40L (TNFSF4)	NM_003326.5	+	CAGTGCACATGCAGGCCTAA
		-	AGCGAGTGAGCACCTAAGAG
OX40L (TNFSF4)	NM_003326.5	+	TTCATCTTCCCTCTACCCAGA
		-	ATCTTGGCCTGGCTGCATTT
OX40 (TNFRSF4)	NM_003327.4	+	ACAAGCCTGGAGTTGACTGT
		-	GTGCTTCCCAGCCAAGGT
IL-2-RA	NM_000417.3	+	CAAGTGACACCTCAACCTGAA
		-	TCCCTGCAGTGACCTGGAA
FOXP3	NM_014009.4	+	ACACTGCCCCTAGTCATGGT
		-	TCCTGGAGGAGTGCCTGTAA
Beta Actin (ACTB)	NM_001101.5	+	GTGACAGCAGTCGGTTGGA
		-	GCATCTCATATTTGGAATGACT
TFRC	M_001128148.3	+	TCGTGTCATGAGAGTGGAGT
		-	CCCCAGAAGACATGTCGGAA
GAPDH	NM_002046.7	+	TGCACCACCAACTGTTAGC
		-	GGCATGGACTGTGGCATGAG

Supplemental materials & methods - Additional procedures

Patients and Tissue Specimens

Tumor samples were obtained from archived surgical specimens collected from 30 patients with stage II–III primary cutaneous melanoma who underwent biopsy or surgical resection at Shamir Medical Center between 2016 and 2023, and further analyzed following approval of the institutional review board (IRB). Informed consent was not required by the committee. Patients with prior systemic therapy or non-cutaneous melanoma were excluded. Clinicopathologic data were retrieved from electronic medical records (Supplementary Table 1 and Fig. S1) and included age, sex, tumor site, Breslow thickness, Clark level, mitotic count, ulceration, sentinel lymph node (SLN) status, recurrence, and survival outcome. Follow-up duration is reported in the Supplement (median, 43 months; range, 12–92).

Multiplex Immunofluorescence (mIF) Staining

FFPE tissue sections (3 µm) were processed manually. Standard protocols were applied for deparaffinization, graded ethanol rehydration, antigen retrieval (10 mM Tris, 1 mM EDTA, pH 9.0, 30 min at 95 °C), and quenching of endogenous peroxidase (3% H_2O_2, 60 min, RT). Immunostaining was performed in three sequential rounds: blocking (10% normal goat serum, 60 min, RT), primary antibody incubation (overnight, 4 °C), poly-HRP–conjugated secondary antibody (60 min, RT), and tyramide-fluorophore detection (5 min, RT; Tyramide SuperBoost kit, Thermo Fisher Scientific, Waltham, MA, USA). Antibodies were stripped between rounds by microwave heating (15 min, 10 mM sodium citrate, pH 6.0). No autofluorescence correction was applied; nonspecific signals were excluded by threshold determination (see below). Slides were counterstained with DAPI and mounted in EverBrite™ Hardset medium (Biotium, Fremont, CA, USA).

Validation of OX40L detection specificity

Specificity of OX40L detection was validated using melanoma cell lines stably transfected with OX40L and matched empty-vector controls processed as paraffin-embedded cell blocks, demonstrating specific staining in transfected cells only. OX40L expression in these cells was independently confirmed at the mRNA and protein levels by RT-qPCR and Western blot, as detailed below and shown in Supplementary Figure S7.

Generation of OX40L-expressing stable melanoma cell clones

The melanoma cell lines Mel624 and Mel14PA were obtained from Dr. Avni (Sheba Medical Center). Cells were stably transfected with an expression vector based on the pEZ-M67 plasmid backbone (GeneCopoeia), harboring the human OX40L (TNFSF4) cDNA (NCBI Gene ID: 7292; NM_003326.5) under the control of a CMV promoter and a hygromycin resistance cassette. Cells transfected with an empty vector lacking a gene insert and the corresponding non-transfected parental cell lines were used as negative controls. Correct insertion of the OX40L cDNA was verified by sequencing. Transfections were performed using lipophilic transfection reagents (Invitrogen, Mirus, Promega) with either native or linearized plasmid DNA. Following 10 days of hygromycin selection, resistant clones were expanded for downstream analyses. OX40L expression was verified by mRNA extraction and RT-PCR separately described in supplemental materials and methods.

Protein extraction and Western blot analysis

OX40L protein expression was verified by using anti-OX40L antibody (clone D6X2D; Cell Signaling Technology) designated for Western blot. Cells were detached using non-enzymatic cell dissociation solution (BI 03-071-1B) or lysed in buffer containing 50 mM Tris-HCl (pH 7.6), 150 mM NaCl, 0.5% NP-40, and 5 mM EDTA, supplemented with protease and phosphatase inhibitors (Protease Inhibitor Cocktail, 50×, G6521; Promega). Lysates were incubated on ice for 30 min and clarified by centrifugation at 18,000 rpm for 30 min at 4 °C. Protein concentration

was determined by Bradford assay, and equal amounts of protein (50 µg) were mixed 1:1 with 2× Laemmli sample buffer (S3401-10VL, Sigma-Aldrich). Samples were resolved on 4–12% ExpressPlus PAGE gradient gels (M42010, GenScript) using Tris-MOPS-SDS running buffer (M00138, GenScript) and transferred to nitrocellulose membranes. Membranes were blocked in 5% bovine serum albumin (BSA) in Tris-buffered saline containing 0.1% Tween-20 (TBST) and incubated with rabbit monoclonal anti-OX40L antibody (clone D6X2D; Cell Signaling Technology) at 1:1000 dilution. After washing, membranes were incubated with HRP-conjugated goat anti-rabbit secondary antibody (Cell Signaling Technology, 1:1000). β-Actin was detected using sequential incubation with mouse monoclonal anti-β-actin antibody (clone 8H10D10; Cell Signaling Technology, 1:1000) followed by HRP-conjugated goat anti-mouse antibody (Cell Signaling Technology, 1:5000). Signals were visualized using enhanced chemiluminescence (ECL), and images were acquired using a digital imaging system.

Immunocytochemistry

Cells were seeded onto sterile glass coverslips and allowed to adhere under standard culture conditions. Cells were fixed in cold methanol (–20 °C) for 10 min and rehydrated in PBS. Non-specific binding was blocked in PBS containing 5% bovine serum albumin (BSA) and 0.1% Tween-20. Coverslips were incubated with IHC-specific rabbit monoclonal anti-OX40L antibody (clone D6K7R; Cell Signaling Technology #59036) at 1:100 dilution, followed by Alexa Fluor® 488–conjugated goat anti-rabbit IgG secondary antibody (Abcam #ab150081, pre-adsorbed, 1:500). Nuclei were counterstained using DAPI-containing antifade mounting medium. Images were acquired using a fluorescence microscope (Lionheart, Agilent) under identical acquisition settings across conditions.

Cell block preparation, immunohistochemistry and immunofluorescence

Cell block preparation was performed according to Koh CM et al. (Methods Enzymol. 2013;533:249–255). Briefly, 1.6×10^7 cells were detached using a non-enzymatic 2 mM

EDTA/EGTA buffer and fixed in ice-cold 70% ethanol for 16 h at 4 °C. After washing, cell pellets were resuspended in 100 µL PBS at room temperature and transferred into pre-set 0.5 mL tubes containing 130 µL molten 2% agarose maintained at 42 °C. Samples were rapidly mixed and allowed to solidify at room temperature or 4 °C for 5–15 min. Solidified agarose-cell plugs were placed in cassettes, stored in a humid chamber at 4 °C, fixed overnight in 10% formaldehyde at room temperature, and processed for paraffin embedding using an automated tissue processor (Logos, Gamidor Diagnostics, Switzerland). Cell block sections (3 µm) were prepared and mounted onto glass slides. Immunohistochemistry was performed using the ultraView Universal DAB Detection Kit on the Ventana Ultra automated immunostainer according to the manufacturer's instructions (Ventana Medical Systems, Oro Valley, AZ, USA). Immunofluorescence staining of FFPE cell block sections was performed using the same protocols, reagents, and amplification strategies as described for FFPE melanoma tumor sections in the main manuscript and the corresponding Supplemental Materials and Methods. Rabbit monoclonal anti-OX40L antibody (clone D6K7R, IHC specific; Cell Signaling Technology #59036) was used at a dilution of 1:100 for both IHC and IF procedures.

Fluorescence Microscopy and Image Analysis

Slides were imaged on a Biotek Lionheart FX fluorescence microscope using GEN5 PRIME software (Agilent, Santa Clara, CA, USA). For each tumor, 5–15 regions of interest (ROIs; 3 mm^2 each) were manually selected. Cells were segmented automatically using GEN5 PRIME. Regions with excessive autofluorescence or strong OX40L signal in skin-associated structures (e.g., blood vessels, as noted in the Results) were manually excluded using the plug function. Positive signal thresholds (10,000–30,000 mean pixel intensity per channel) were applied to exclude background fluorescence and were defined based on three randomly selected images per staining set. Object size parameters were set between 2 and 30 µm depending on cell type.

For marker co-expression analysis, one marker was defined as the primary mask, with secondary markers quantified within expanded object boundaries. Proteins were considered co-expressed when detected within ≤2 µm of each other, reflecting subcellular-scale proximity consistent with object-based and segmentation-driven multiplex immunofluorescence analyses that assign co-expression only when signals reside w**ithin the same cell compartment.** For intercellular proximity analysis, cells were considered co-localized when detected within ≤20 µm of each other (Fig. S2a), corresponding approximately to one cell diameter and consistent with commonly used thresholds in spatial mIF studies to capture immediate cell–cell neighborhoods compatible with membrane contact or short-range juxtacrine signaling (Dunn et al., 2011; Tsakiroglou et al., 2020; Almekinders et al., 2022; dos Santos Peixoto et al., 2025)

Enrichment of Tregs from Peripheral Blood

PBMCs were isolated from fresh buffy coat obtained by leukapheresis from an anonymous healthy donor (Blood Bank Laboratories, Sheba Medical Center, Tel Hashomer, Israel). The 10 mL leukocyte-enriched fraction was diluted 1:4 with PBS, layered onto Lymphoprep (#07801, STEMCELL Technologies, Vancouver, Canada), and centrifuged (750 ×g, 30 min, RT, brake off). PBMCs were collected, washed, and resuspended in PBS + 2% FBS.

Tregs were enriched using the EasySep™ Human $CD4^{+}CD127^{low}$ $CD25^{+}$ Regulatory T Cell Isolation Kit (#18063, STEMCELL Technologies, Vancouver, Canada). Each batch began with 5×10^{7} PBMCs and yielded ~1.6×10^{6} enriched Tregs; three parallel batches were processed from the single donor. Post-enrichment analysis showed >85% $CD4^{+}CD25^{+}$ cells among live cells; $Foxp3^{+}$ frequency was ~30%, consistent with known limitations of Foxp3 detection by flow cytometry.

Treg Expansion in Culture

Enriched Tregs were cultured in ImmunoCult™-XF medium (#100-0956, STEMCELL Technologies, Vancouver, Canada) supplemented with antibiotics (1% Penicillin–Streptomycin–Amphotericin B; Biowest, Nuaillé, France) and 500 IU/ml recombinant human IL-2 (Sigma-Aldrich, St. Louis, MO, USA). Cells were plated at 1×10^6/ml and stimulated with ImmunoCult™ Human CD3/CD28 T Cell Activator (#100-0784, STEMCELL Technologies, Vancouver, Canada). Cultures were maintained for 21 days with medium changes every 2–3 days. Expansion was monitored by serial cell counts and viability measurements.

Flow Cytometry

Cells (10^5–10^6 per test) were washed in PEB buffer (PBS, 0.5% BSA, 2 mM EDTA, pH 7.2), incubated with Viobility Fixable Dye (Miltenyi Biotec, Bergisch Gladbach, Germany; 15 min, RT), and stained with directly conjugated antibodies against CD4, CD25, OX40, and OX40L. Intracellular Foxp3 was detected using the FoxP3 Staining Buffer Set (#130-093-142, Miltenyi Biotec). Data were acquired on a MACSQuant® Analyzer 10 with MACSQuantify™ software v3.02 (Miltenyi Biotec). Compensation was performed with both single-stain and bead controls (#130-104-693, #130-097-900, Miltenyi Biotec). Gating strategy is described in the main Methods. Antibodies and fluorophores are listed in Supplementary Table 3.

RNA Extraction and RT-qPCR

RNA was extracted using the SV Total RNA kit (#Z3100, Promega, Madison, WI, USA). Purity was confirmed by A260/280 ratio >1.8 (NanoDrop; Thermo Fisher Scientific, Waltham, MA, USA). cDNA synthesis was performed with 100 ng RNA using the SuperScript III First-Strand Kit (#18080051, Thermo Fisher Scientific, Waltham, MA, USA). qPCR was performed in duplicate reactions with PerfeCTa SYBR Green FastMix (#95074-012, Quantabio, Beverly, MA,

USA) on a Rotor-Gene 6000 instrument (Corbett, Sydney, Australia). Cycling: 95 °C for 2 min, then 40 cycles of 95 °C for 5 s and 60 °C for 30 s. Expression was normalized to GAPDH, and / or β-actin and TFRC reference genes as specified. Results are reported as $2^{\wedge}–\Delta Ct \times 10^4$. OX40L expression was calculated as the mean of two primer sets targeting distinct loci. Primer sequences and gene accession numbers are provided in Supplementary Table 4.

Statistical Analysis

Statistical analyses were selected a priori based on data structure and level of comparison. ROI-level quantitative comparisons between groups were assessed using two-tailed Student's t-tests. Associations between categorical marker expression patterns at the tumor level were evaluated using χ^2 tests. Linear relationships between continuous variables were assessed using Pearson correlation coefficients. Recurrence-free survival was analyzed using Kaplan–Meier estimates with log-rank testing. These complementary approaches enabled evaluation of quantitative differences, categorical associations, and clinical outcomes using established statistical frameworks, and are detailed in the main Methods section.

Additional methods details

Additional methodological details are available upon request.

Supplemental References

Almekinders, M. M., Bismeijer, T., Kumar, T., Yang, F., Thijssen, B., van der Linden, R., et al. (2022). Comprehensive multiplexed immune profiling of the ductal carcinoma in situ immune microenvironment regarding subsequent ipsilateral invasive breast cancer risk. *Br. J. Cancer* 127, 1201–1213. doi: 10.1038/s41416-022-01888-2

dos Santos Peixoto, R., Miller, B. F., Brusko, M. A., Aihara, G., Atta, L., Anant, M., et al.

(2025). Characterizing cell-type spatial relationships across length scales in spatially resolved omics data. *Nat. Commun.* 16, 350. doi: 10.1038/s41467-024-55700-1

Dunn, K. W., Kamocka, M. M., and McDonald, J. H. (2011). A practical guide to evaluating colocalization in biological microscopy. *Am. J. Physiol. Cell Physiol.* 300, C723-42. doi: 10.1152/ajpcell.00462.2010

Tsakiroglou, A. M., Fergie, M., Oguejiofor, K., Linton, K., Thomson, D., Stern, P. L., et al. (2020). Spatial proximity between T and PD-L1 expressing cells as a prognostic biomarker for oropharyngeal squamous cell carcinoma. *Br. J. Cancer* 122, 539–544. doi: 10.1038/s41416-019-0634-z
