# Supplementary material for: Prognostic immunological implications of OX40L expression in the tumor microenvironment of melanoma
Source: Front Immunol. 2026 Jan 23;17:1745742. doi: 10.3389/fimmu.2026.1745742 (PMC12876231; doi:10.3389/fimmu.2026.1745742)
Supplement: Supplementary file 2 [file Image1.pdf]

Supplemental Figures

Table of content

Figure S1 A-E $OX40L^+$ cell distribution in melanoma and skin

Figure S2 Measurements of co-localization events

Figure S3 A-I $OX40L^+$ cell phenotyping in melanoma tumors

Figure S4 A-E $OX40L^+$ Treg in melanoma tumors and peripheral blood

Figure S5 A-B Immune checkpoint expression in OX40L+ and OX40+ Treg

Figure S6 A-B $OX40L^+$ APC and association with recurrence

Figure S7 A-F Validation of OX40L antibody specificity

Supplemental Figure S1

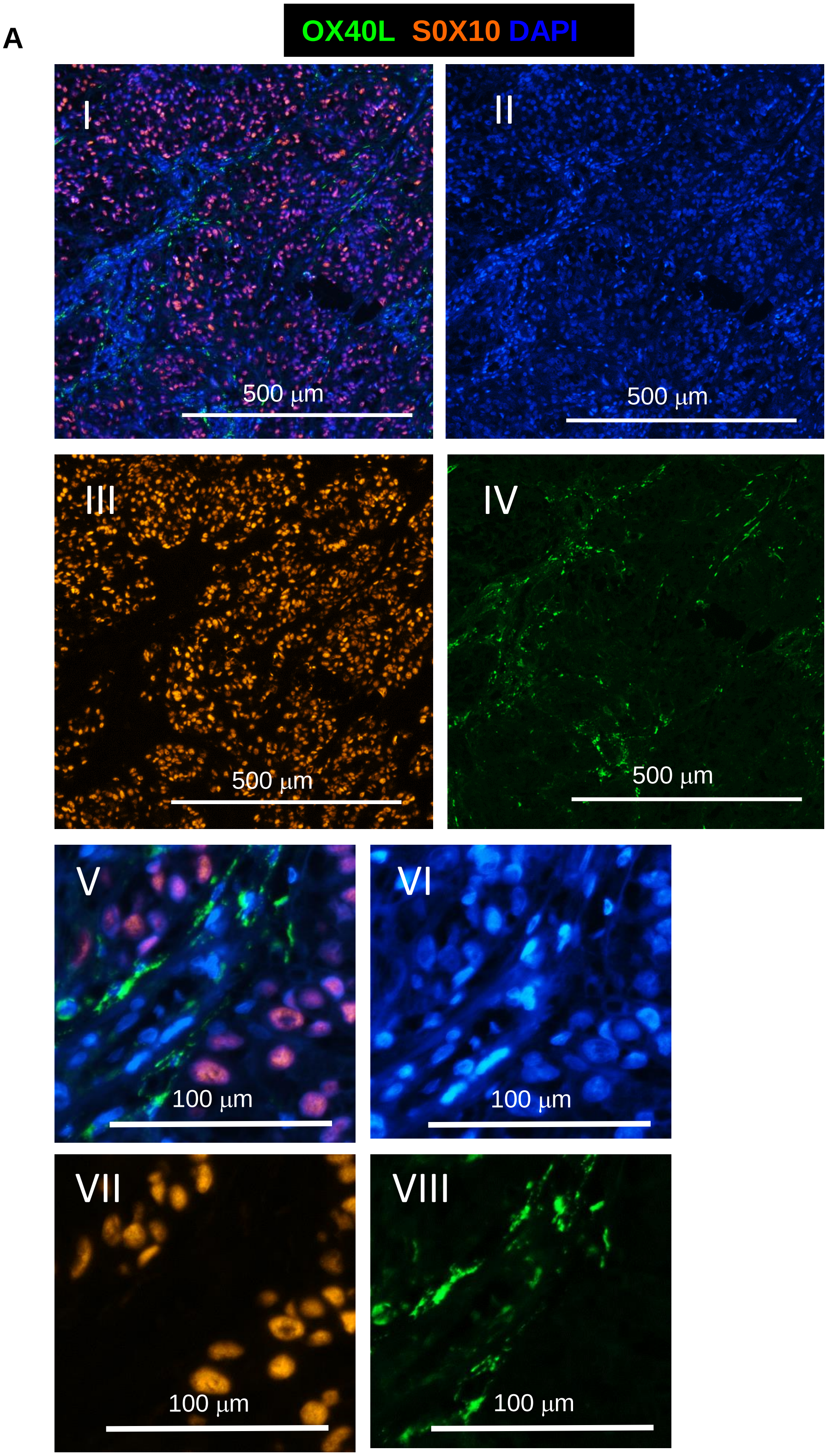

Supplemental Figure S1

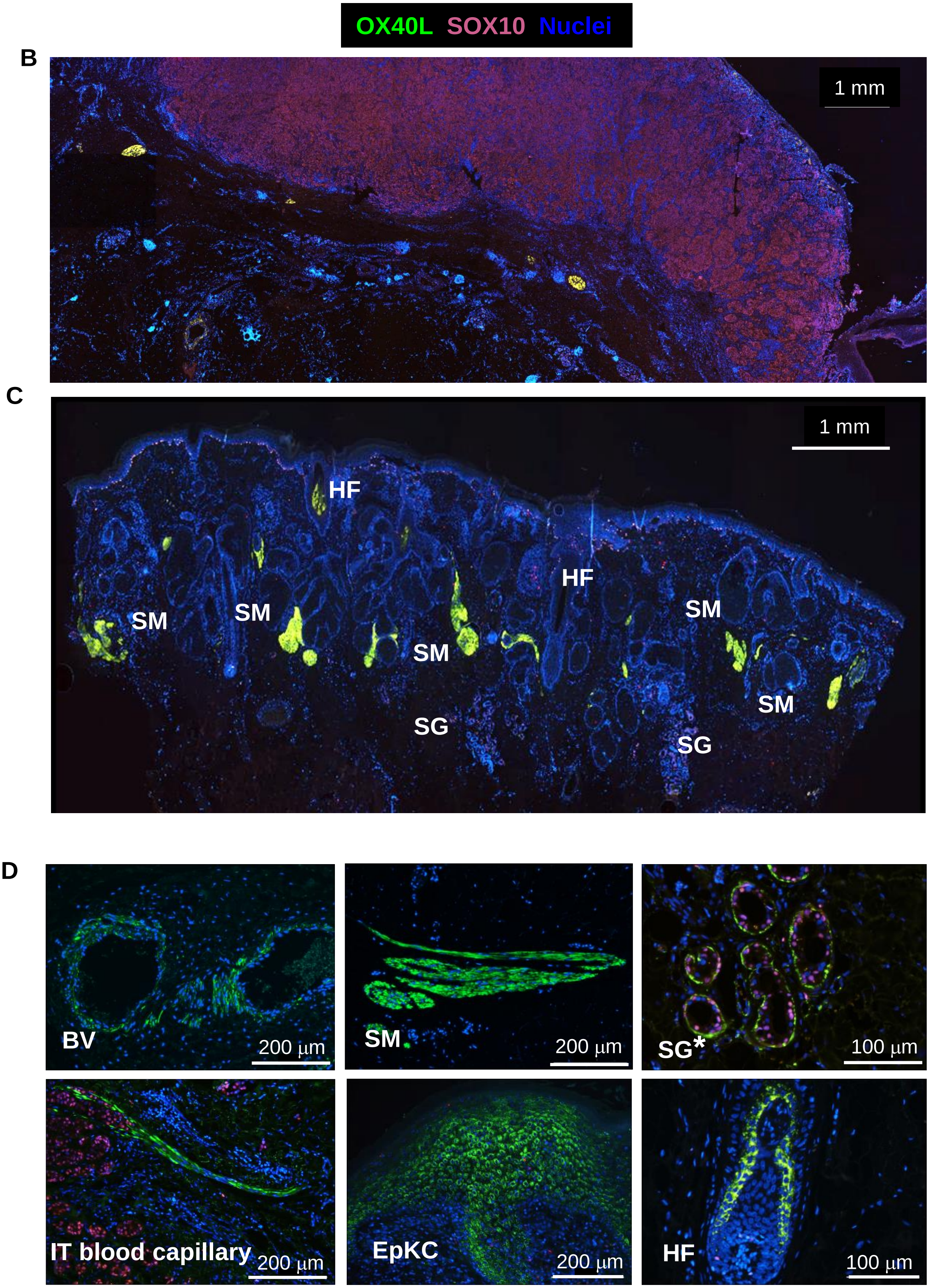

Supplemental Figure S1

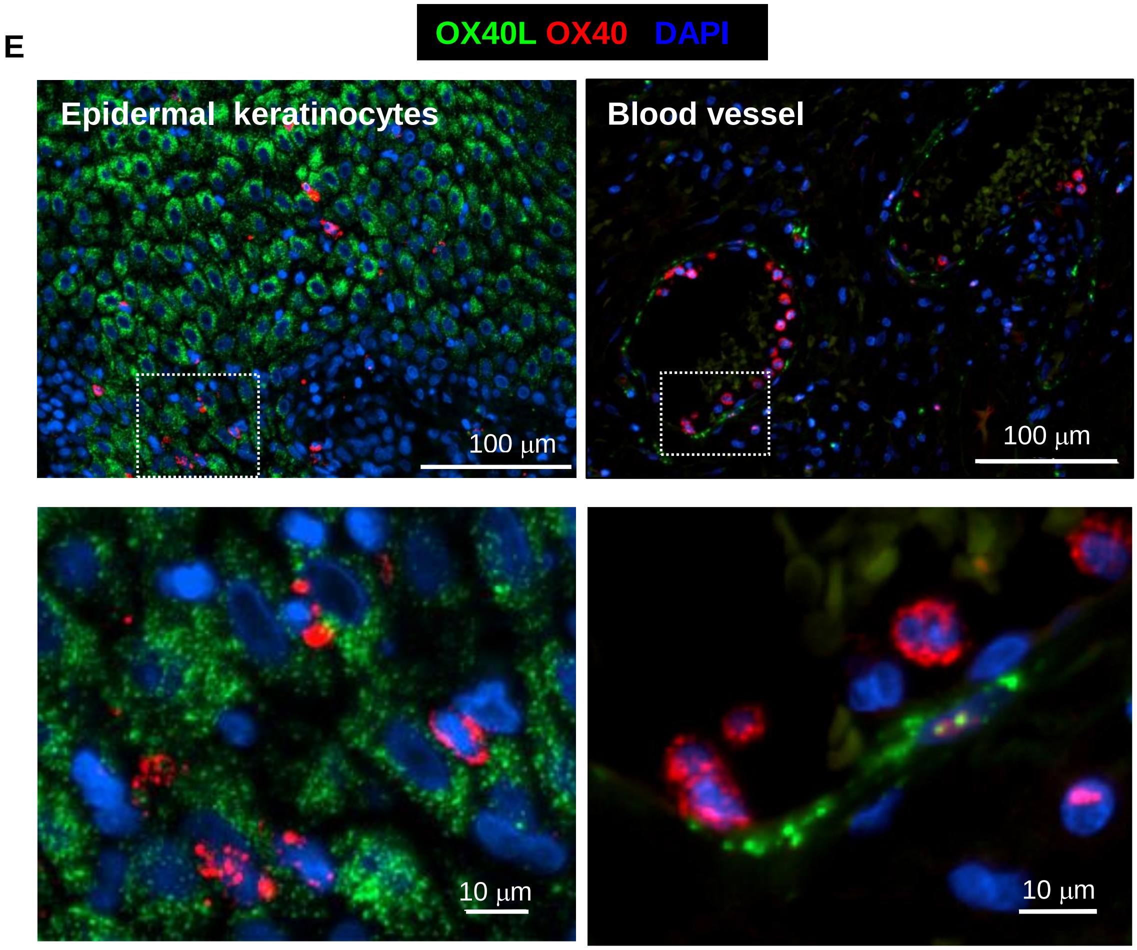

Fig. S1 OX40L⁺ cell distribution in melanoma and skin.

(a) OX40L⁺ cells (green) with elongated morphology forming chain-like patterns among stromal cells (DAPI, blue), compartmentalizing SOX10⁺ melanoma nests (orange/pink). The top-right image (I) is also displayed in separated color channels (II–IV), with larger magnifications of a selected region shown below (V–VIII).

(b–d) Full-section mIF images of melanoma tissue (b) and adjacent normal skin (c) highlighting OX40L⁺ structures in blood vessels endothelium (BV), smooth muscle (SM), sweat glands (SG), and hair follicle keratinocytes (HF). *Note: SOX10 is physiologically expressed in sweat glands and should not be misinterpreted as melanoma signal in these regions.

(e) Co-localization of OX40L⁺ epidermal keratinocytes (left) and OX40L⁺ vascular cells (right) with OX40⁺ cells (red, likely T cells). Higher magnification views of the areas highlighted in white-dashed squares are shown below.

Supplemental Fig. S3 OX40L$^+$ cell phenotyping in melanoma tumors

Representative mIF images showing the diversity of OX40L-expressing cell types in melanoma. All panels include both broader views and magnified, color-separated images.

A

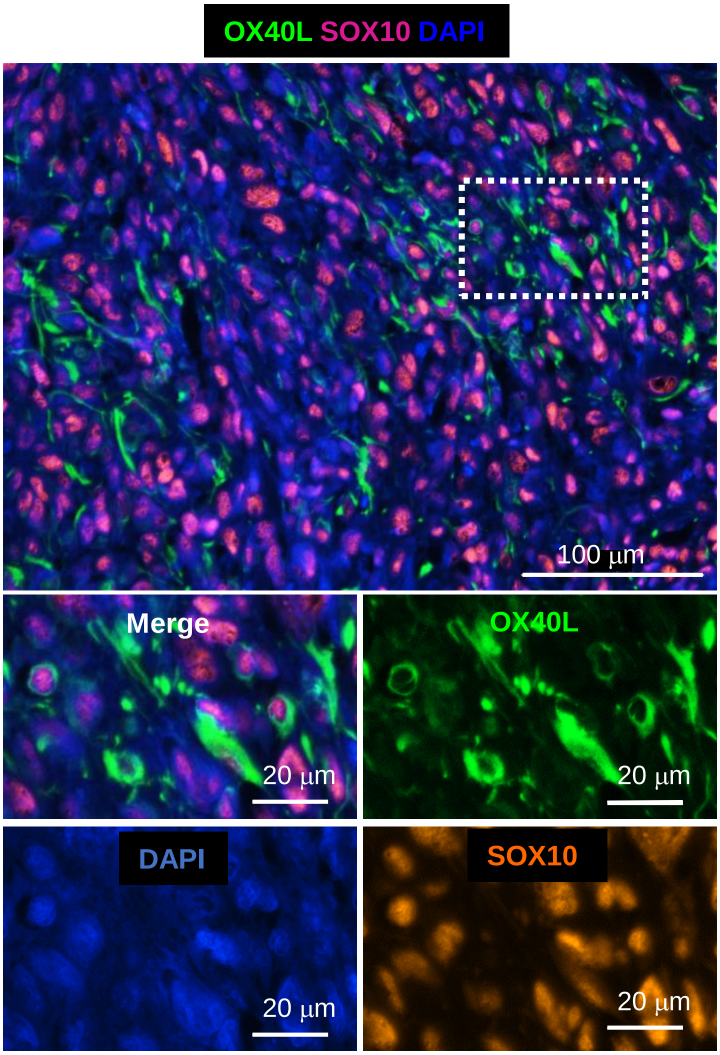

Fig. S3 OX40L$^+$ cell types in melanoma

(a) SOX10$^+$ melanoma cells (orange/magenta), some elongated, expressing OX40L on their membranes (green). This tumor had widespread expression of OX40L on melanoma cells.

Supplemental Figure S3

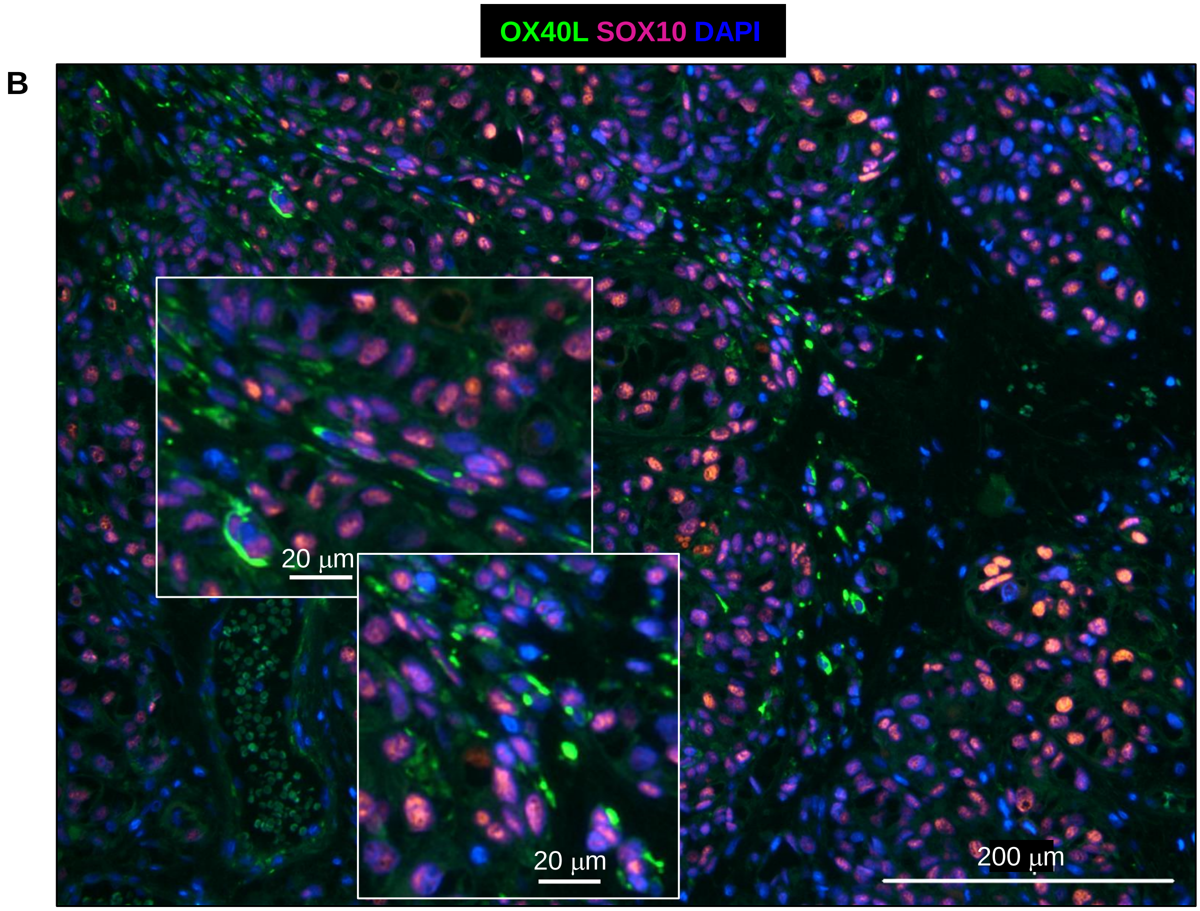

Fig. S3 OX40L⁺ cell types in melanoma

(b) Chain-like arrangements of elongated SOX10⁺ (magenta) OX40L⁺ (green) melanoma cells within tumor parenchyma.

Supplemental Figure S3C-E

C

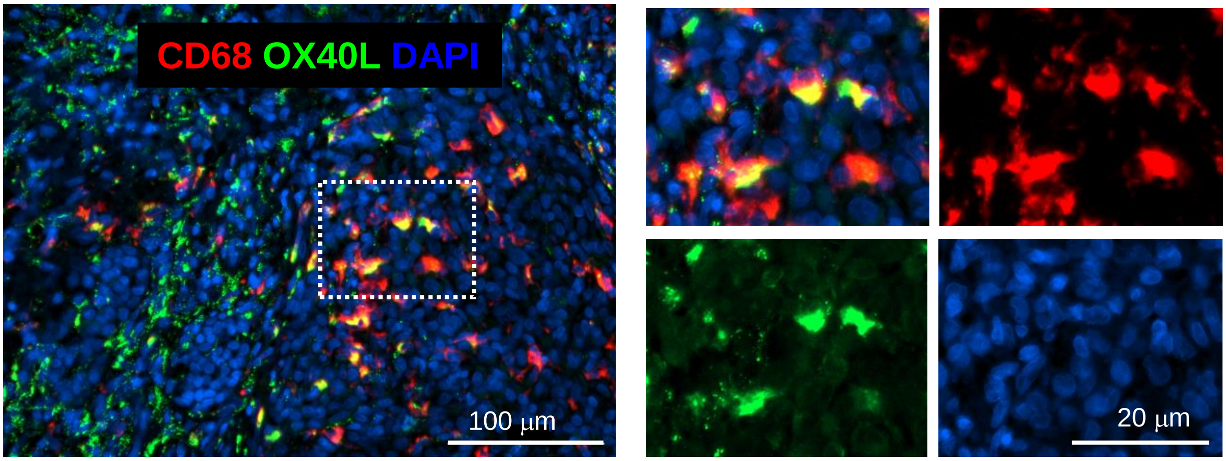

D

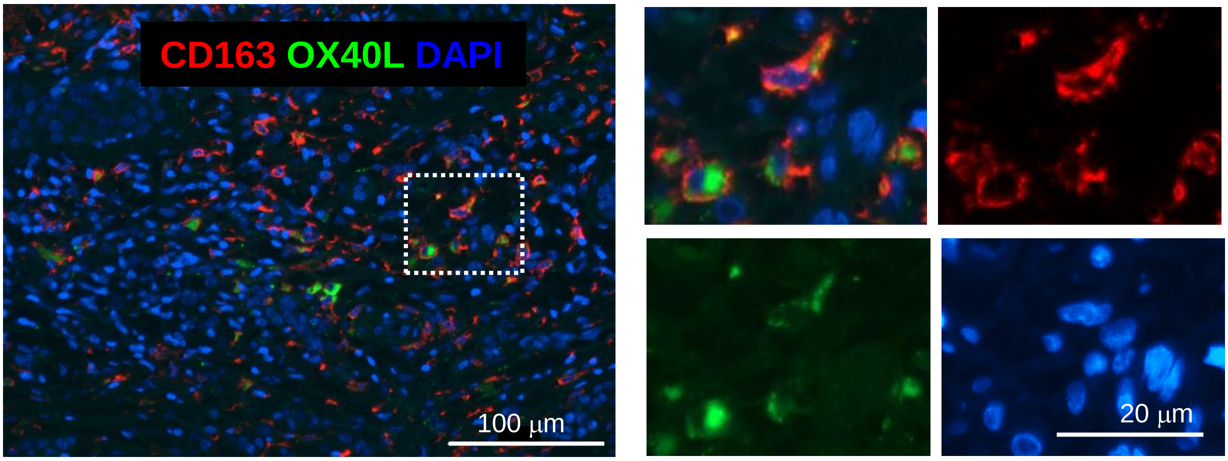

E

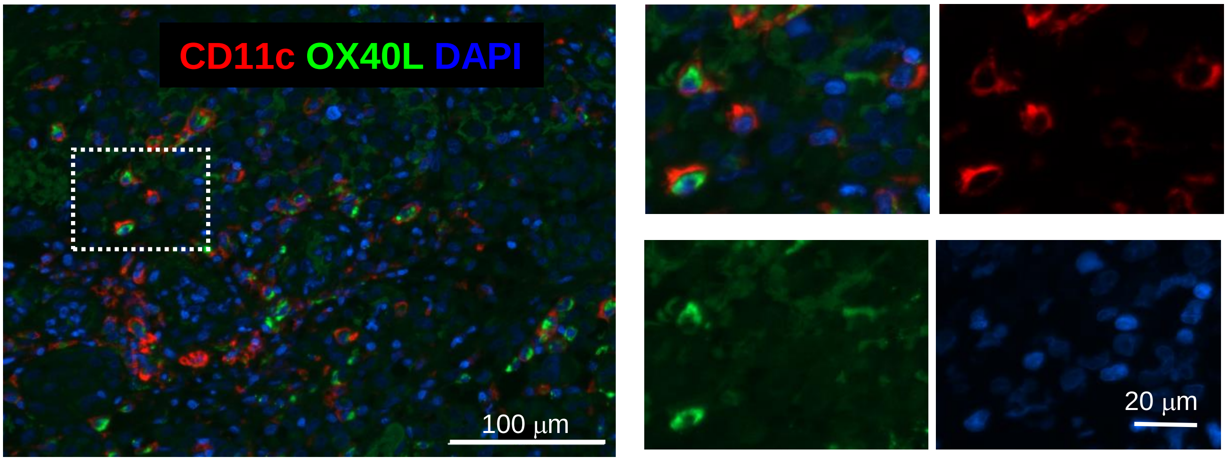

Fig. S3 OX40L^{+} cell types in melanoma

(c–e) Macrophages (CD68, CD163) and dendritic cells (CD11c) expressing OX40L Higher magnification views of the area highlighted by white-dashed squares are shown on the right in separate colors. Scale bars on the right refer to all corresponding color-split images.

Supplemental Figure S3

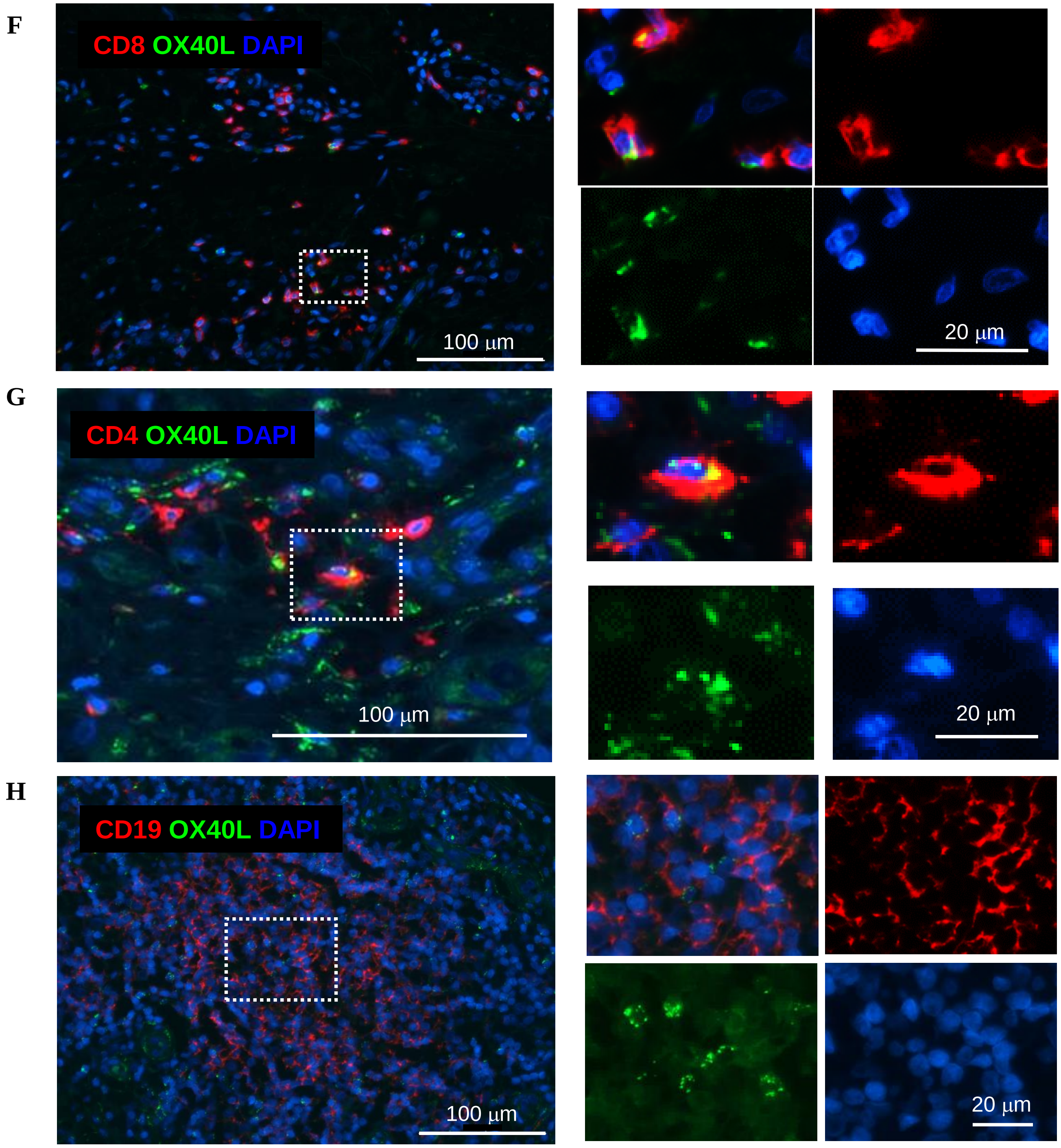

Fig. S3 OX40L^{+} cell types in melanoma

(f–h) T cells (CD4, CD8) and B cells (CD19) expressing OX40L. Higher magnification views of the area highlighted by white-dashed squares are shown on the right in separate colors. Scale bars on the right refer to all corresponding color-split images.

Supplemental Figure S3

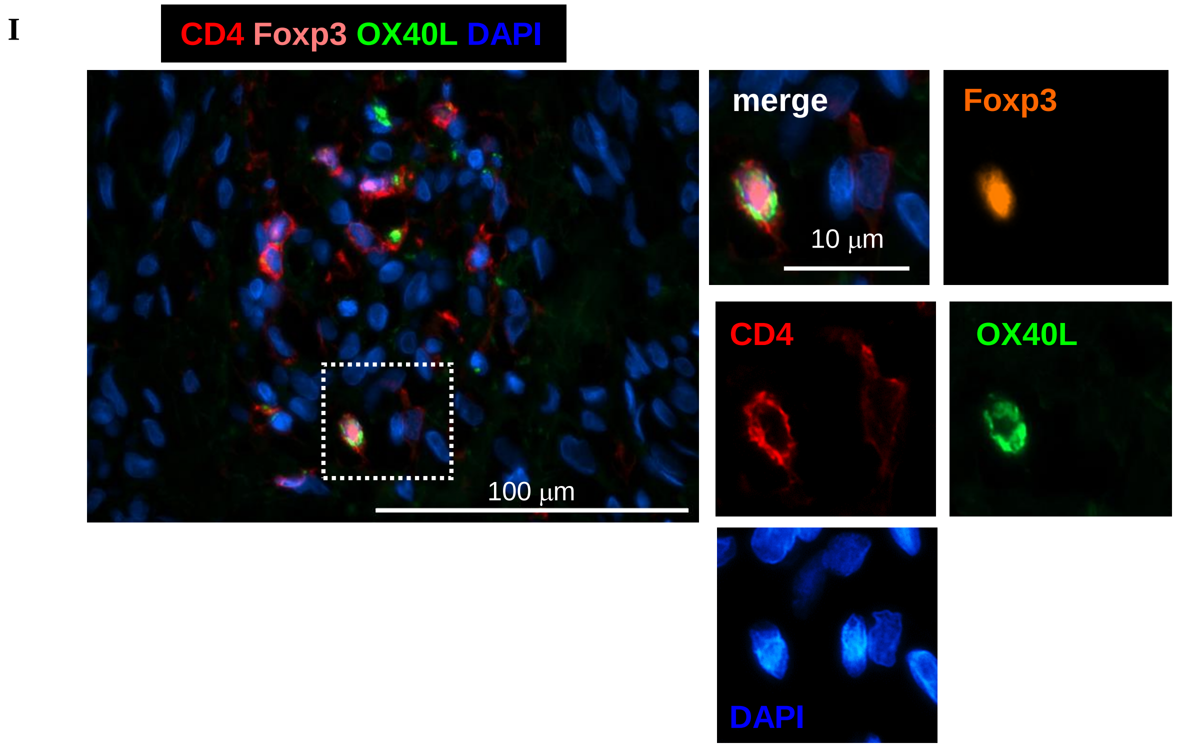

Fig. S3 OX40L^{+} cell types in melanoma

(i) Foxp3^{+}CD4^{+} regulatory T cells (Tregs) expressing OX40L. Higher magnification views of the area highlighted by white-dashed square is shown on the right in separate colors. Scale bar on the right refers to all corresponding color-split images.

Supplemental Figure S4 OX40L$^+$ Treg in melanoma tumors and peripheral blood

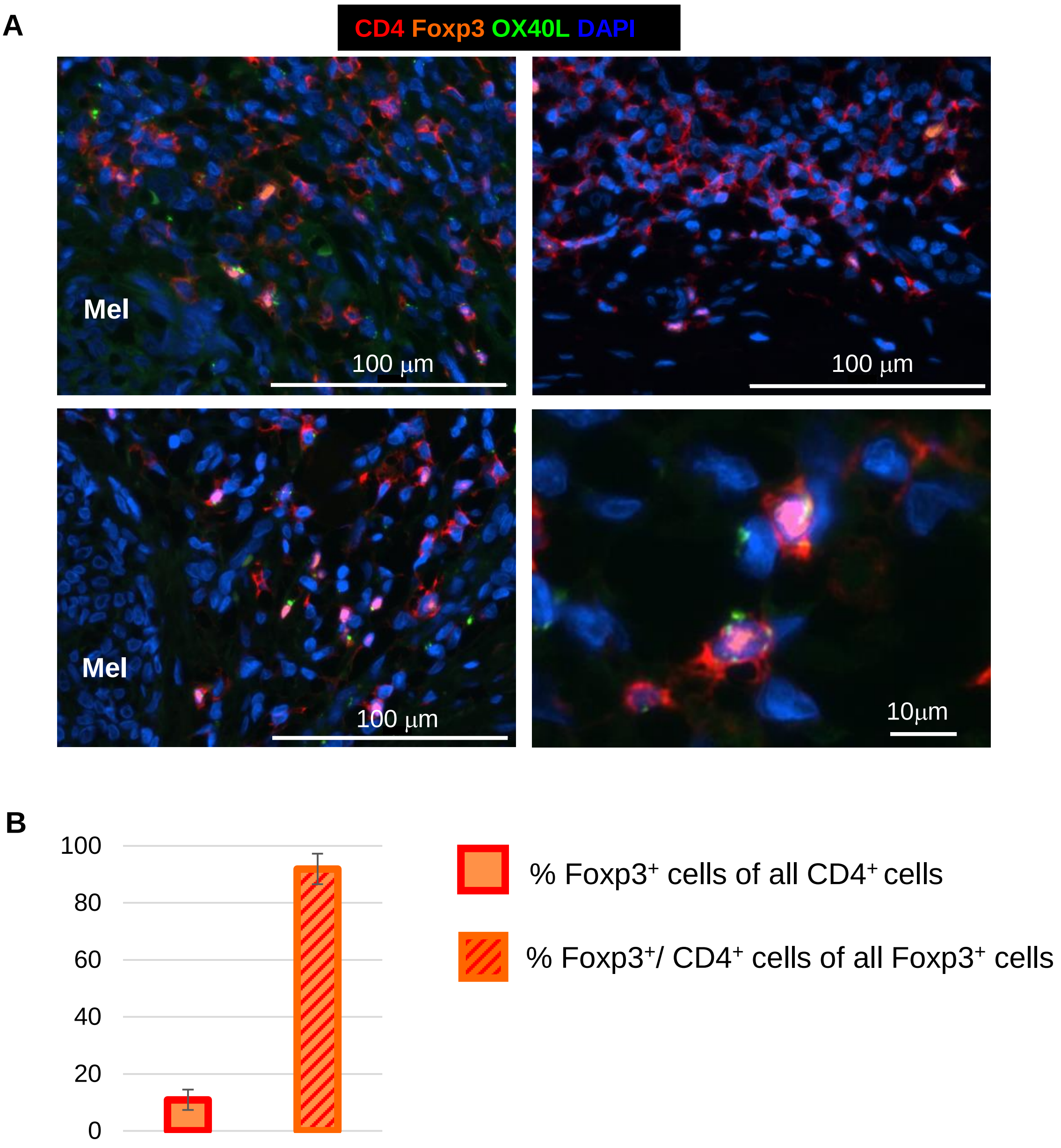

Fig. S4 **CD4–Foxp3 co-expression in Tregs expressing OX40 and/or OX40L**

(a) Representative images of intratumoral CD4$^+$Foxp3$^+$ Tregs expressing OX40L. Foxp3 stains Treg nuclei in orange or in magenta when overlaid with DAPI.

(b) Quantitative analysis showing that ~10% of CD4$^+$ cells are Foxp3$^+$ Tregs, and 92% of Foxp3$^+$ cells co-express CD4 (mean ± SD; n = 3 tumors, 5–20 ROIs/tumor; ROI size = 3 mm²).

Supplemental Figure S4

C

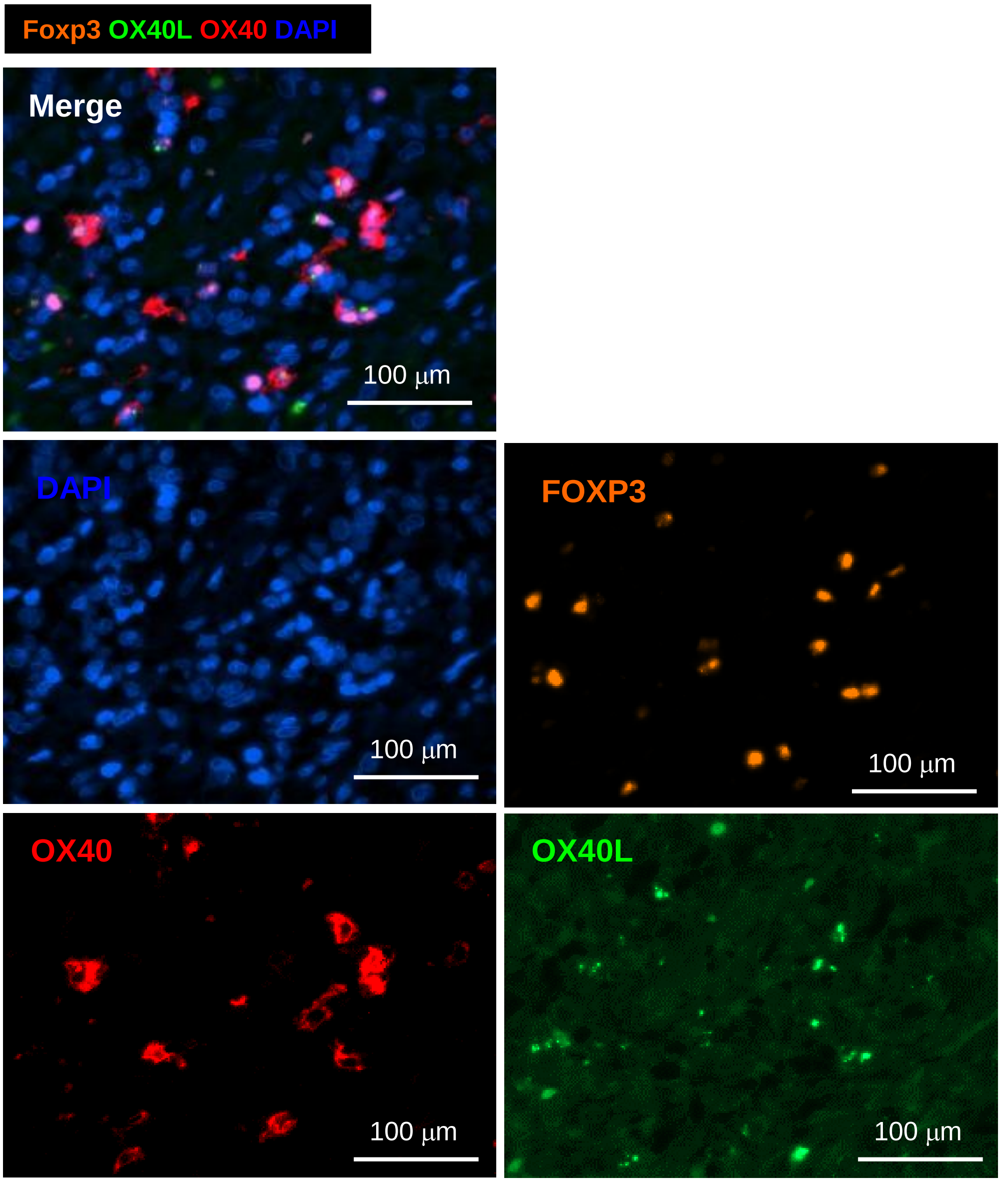

Fig. S4 (c) Foxp3$^+$ Tregs expressing OX40L (green), OX40 (red), both (yellow), or neither. Foxp3 stains Treg nuclei in orange or in magenta when overlaid with DAPI. The image above is shown in separate colors below.

Supplemental Figure S4

D

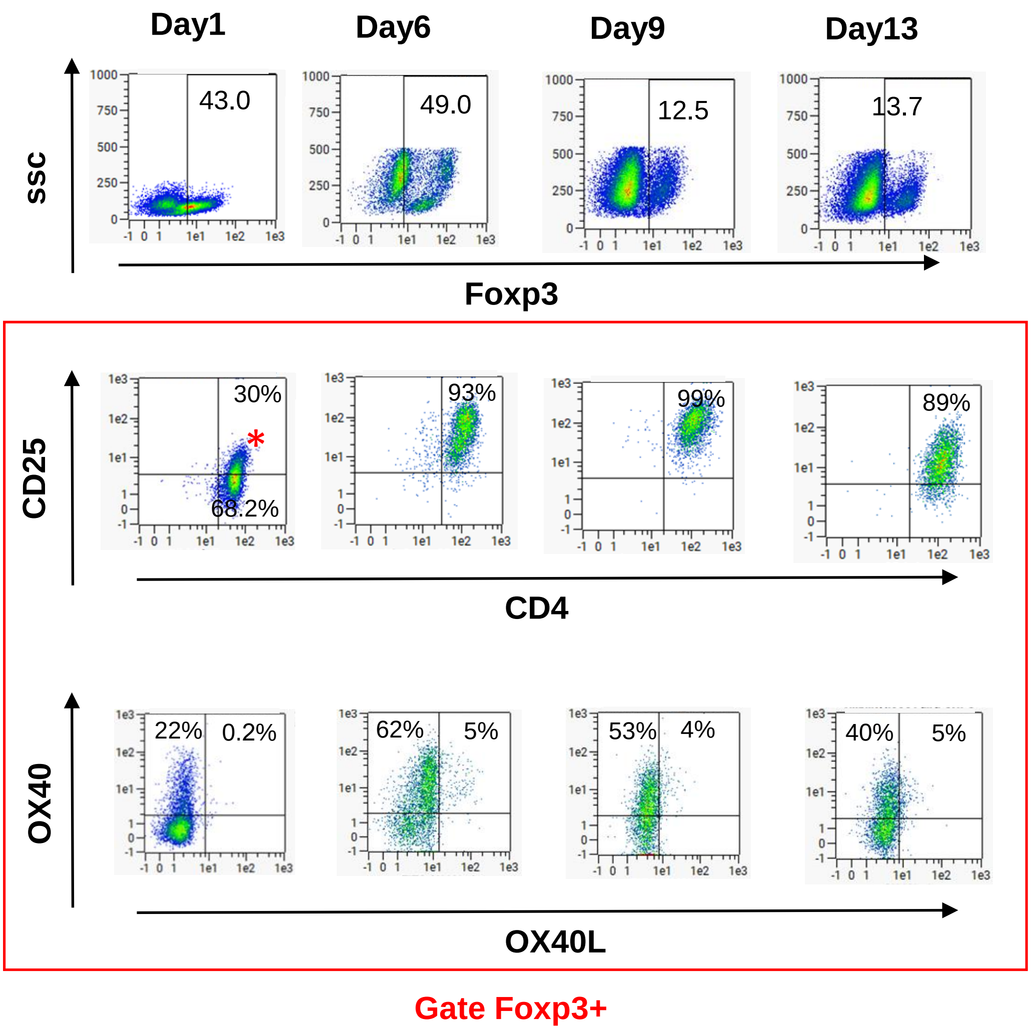

E

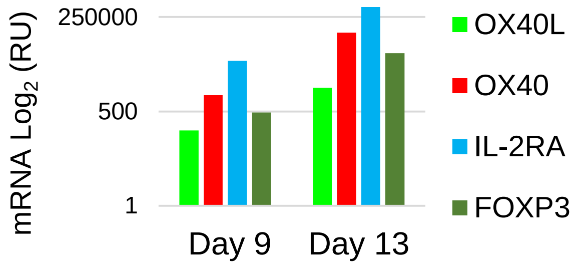

Fig. S4 CD4–Foxp3 co-expression and OX40/OX40L phenotyping in Tregs

(d) Flow cytometry analysis of blood-derived regulatory T cells. $CD4^{+}CD25^{+}CD127^{-}$ Tregs were isolated from the peripheral blood of a healthy donor, enriched by magnetic bead separation, and cultured with IL-2 and CD3/CD28 stimulation. Representative flow cytometry plots from days 1, 6, 9, and 13 show the proportion of $Foxp3^{+}$ cells among total live singlet cells (upper panel) and the proportions of $CD4^{+}/CD25^{+}$ cells and $OX40^{+}/OX40L^{+}$ cells within the $Foxp3^{+}$-gated population (lower panels), with gating defined as described in the Methods.**(e)** RT-PCR analysis of the total Treg-enriched blood-derived population on days 9 and 13 of culture, showing mRNA levels for OX40L, OX40, IL-2RA (CD25), and Foxp3. Values were normalized to GAPDH, β-actin, and TFRC, and are expressed as relative units (RU).

Fig. S5 Immune checkpoint expression in OX40L+ and OX40+ Treg

A

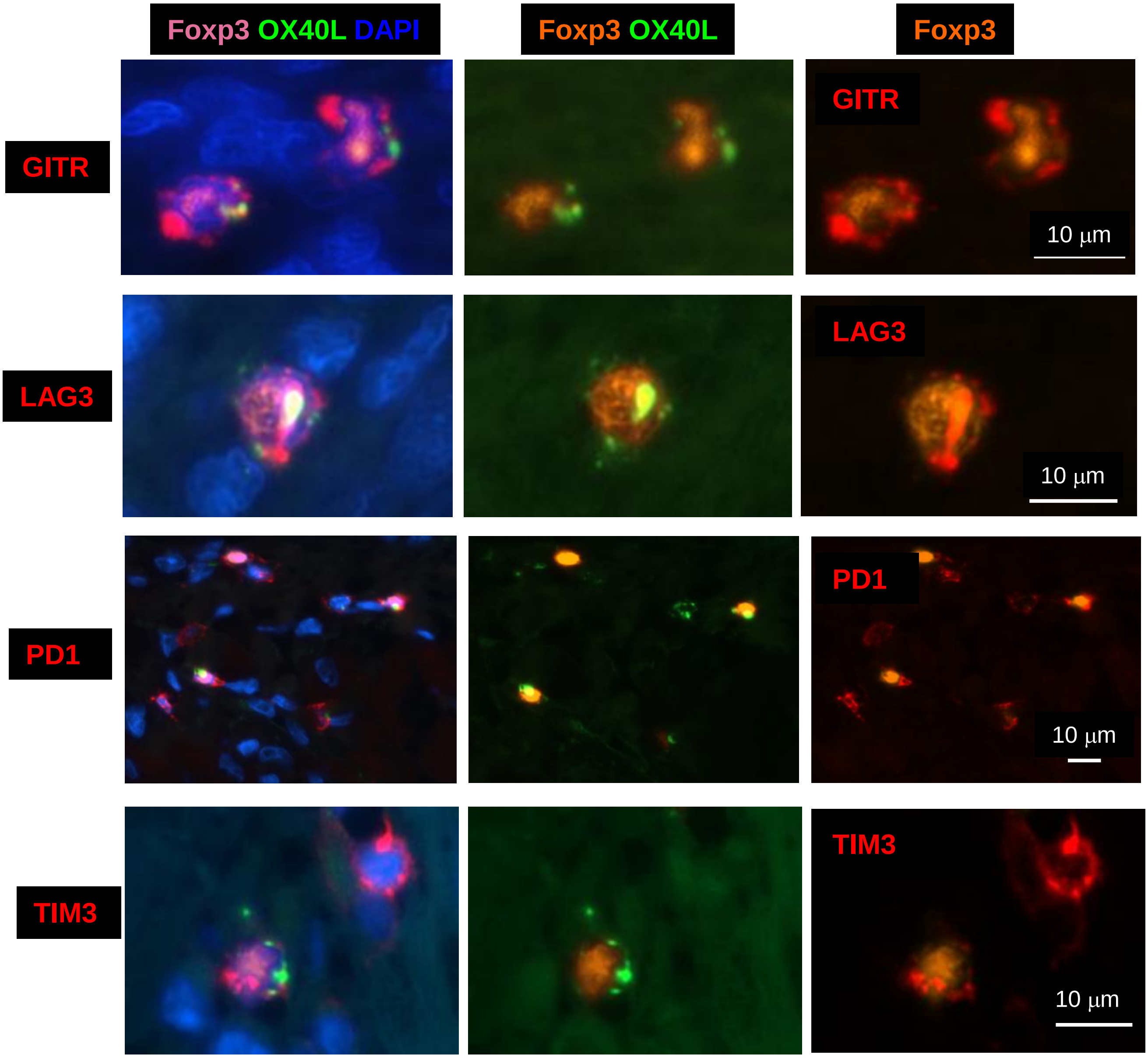

Fig. S5. Immune checkpoint (ICP) expression in OX40L$^+$ and OX40$^+$ Tregs

(a) Representative mIF images showing OX40L (green) co-expressed with GITR, LAG3, PD1, or TIM3 (red) in Foxp3$^+$ Tregs (orange alone or magenta when over-laid with DAPI) and DAPI (blue). Each panel of three images share the same scale bar shown on the right.

Fig. S5

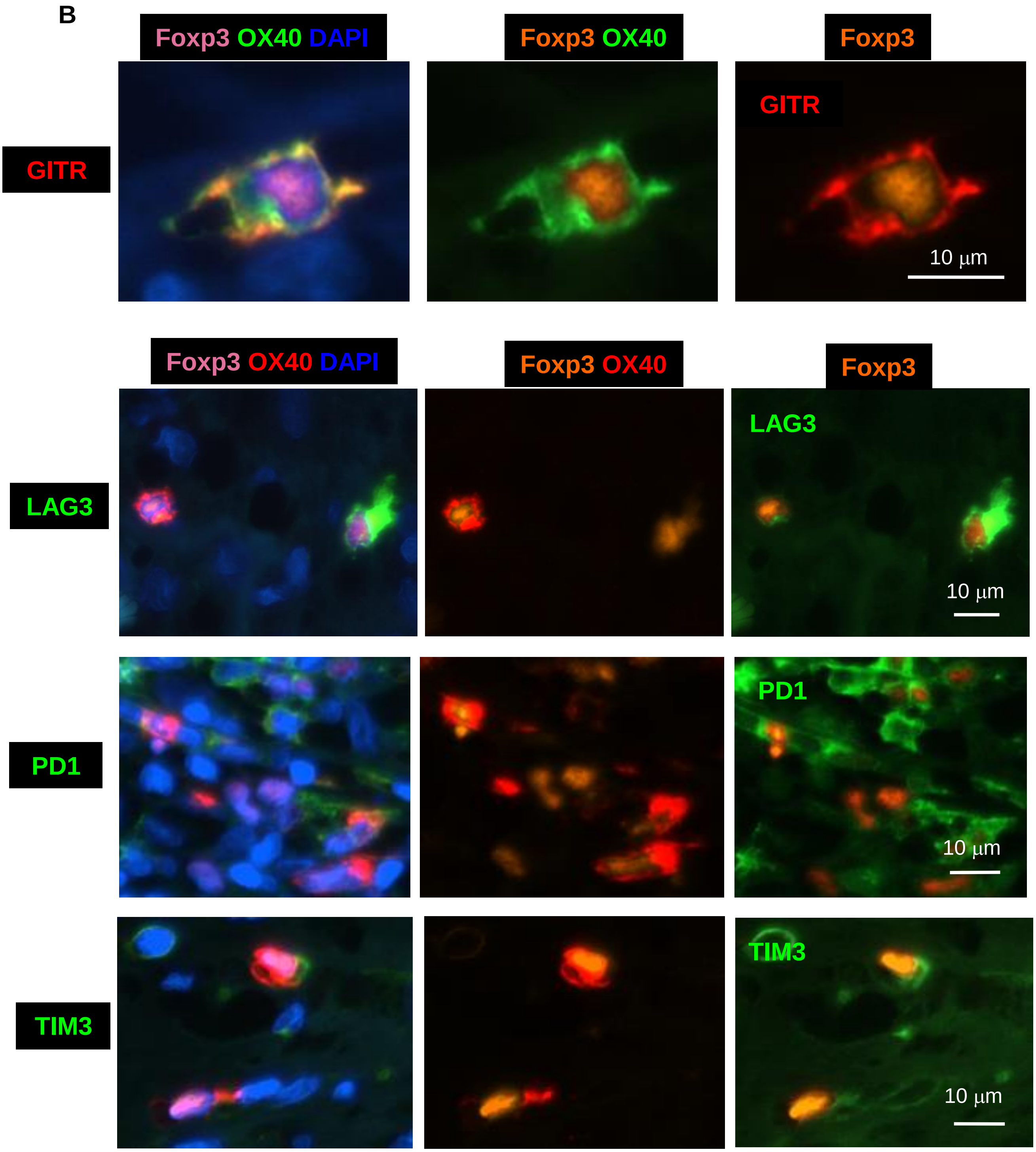

Fig. S5 Immune checkpoint (ICP) expression in OX40L⁺ and OX40⁺ Tregs

(b) Representative mIF images showing OX40 (red) co-expressed with LAG3, PD1, or TIM3 (green). For GITR co-staining, OX40 (green) and GITR (red). Foxp3⁺ Treg nuclei were stained orange alone or magenta when over-laid with DAPI (blue). Each panel of three images share the same scale bar shown on the right.

Fig. S6

A

Patient No.	Time follow up (months)	Censor	Incidence (Recurrence)	Marker % OX40L$^+$ cells in CD163$^+$/CD11c$^+$	Marker group H>, L< median (4.7%)	Tumor Depth (mm)
P1	8	0	NO	1.0	L	6
P2	61	0	NO	1.5	L	4
P3	11	0	NO	3.0	L	5.5
P4	22	0	NO	4.0	L	4
P5	26	0	NO	5.0	H	5
P6	22	0	NO	6.0	H	4.5
P7	42	0	NO	8.0	H	4
P8	25	0	NO	9.0	H	15
P9	37	0	NO	10.0	H	10
P10	58	0	NO	12.0	H	15
P11	10	0	NO	16.0	H	1
P12	24	1	YES	1.0	L	7
P13	16	1	YES	1.5	L	1.5
P14	1	1	YES	2.0	L	10
P15	26	1	YES	2.3	L	5.5
P16	31	1	YES	3.6	L	6
P17	14	1	YES	4.0	L	5
P18	22	1	YES	4.0	L	5
P19	40	1	YES	4.7	H	4
P20	35	1	YES	5.0	H	14
P21	72	1	YES	7.0	H	1.2
P22	8	1	YES	15.0	H	13

Fig. S6

(a) Data table of 22 tumors, listing recurrence status, follow-up time, percentage of OX40L$^+$ cells within the CD163$^+$/CD11c$^+$ myeloid compartment, classification by low (< median 4.7%, L) or high (> median 4.7%, H) prevalence, and tumor depth.

Fig. S6

B

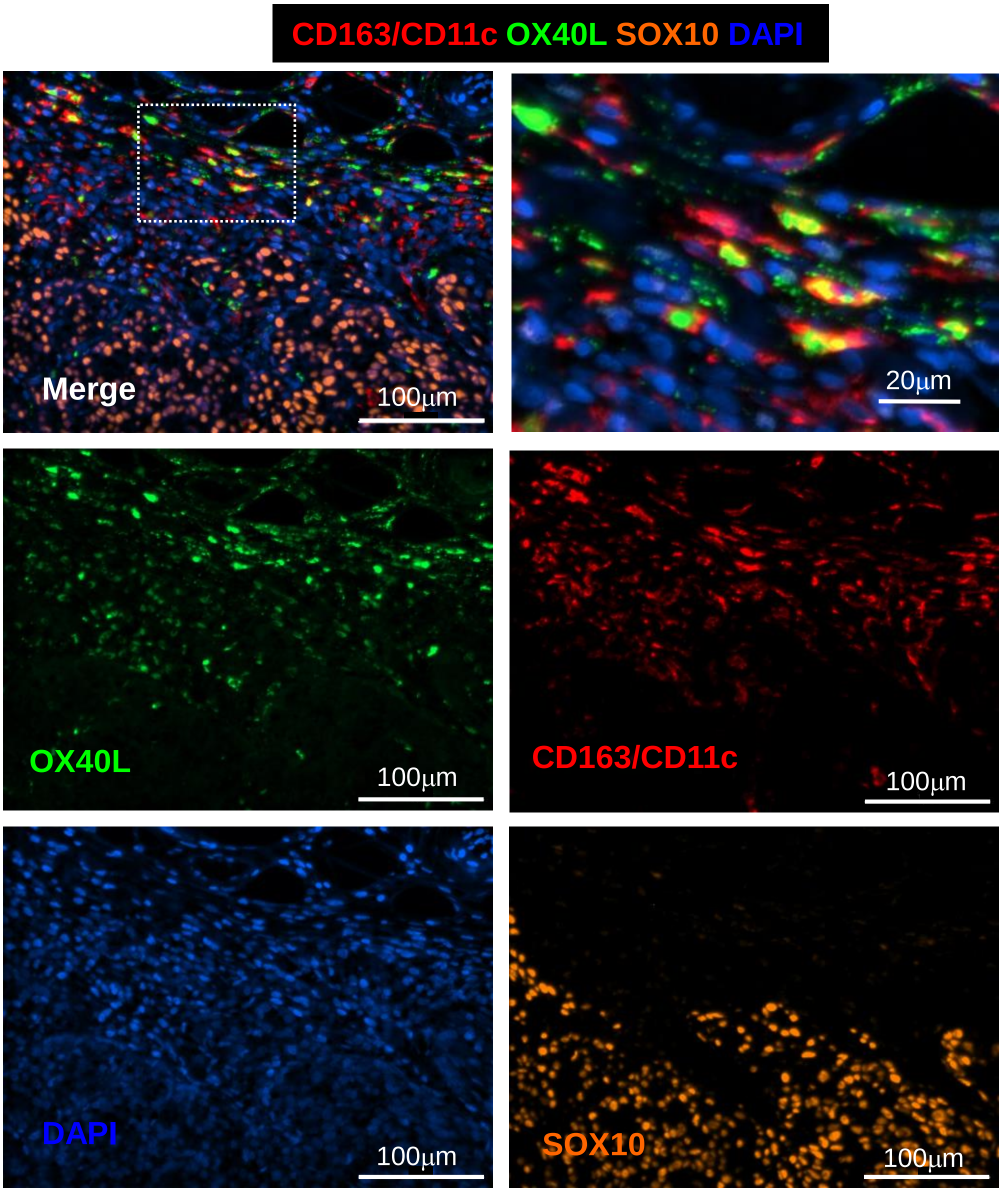

Fig. S6

(b) Representative mIF images showing co-expression of OX40L (green) with myeloid markers CD163/CD11c (red) in melanoma. SOX10 (orange) highlights melanoma nuclei and DAPI (blue) marks all nuclei. Yellow indicates overlap of OX40L with myeloid markers.

Supplemental Fig. S7 – Validation of OX40L antibody specificity

A

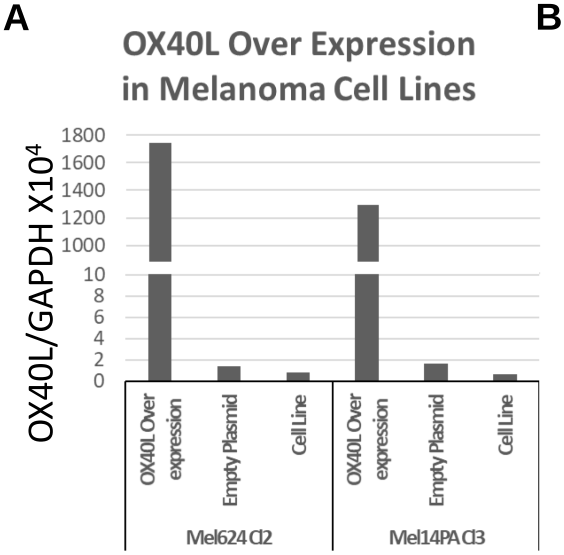

B

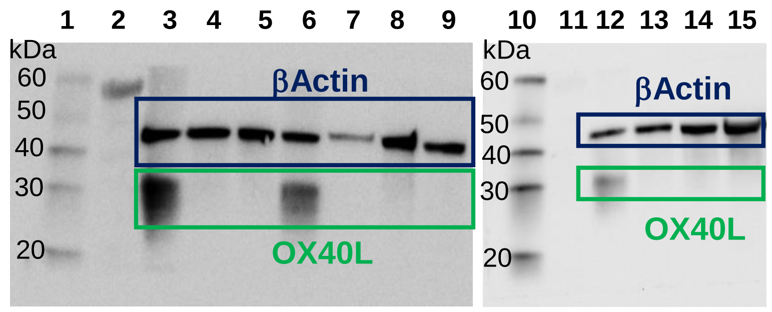

1 Fluorescent Marker, **2** Color Marker, **3** Mel624/OX40L Cl2, **4-5** Mel624/Empty Plasmid Cl2, **6** Mel624/OX40L Cl3, **7-8** Mel624/Empty Plasmid Cl3, **9** Mel624 parental cells, **10** Fluorescent Marker, **11** Color Marker, **12** Mel14PA/OX40L Cl3, **13-14** Mel14PA/ Empty Plasmid Cl3, **15** Mel14PA parental cells

C

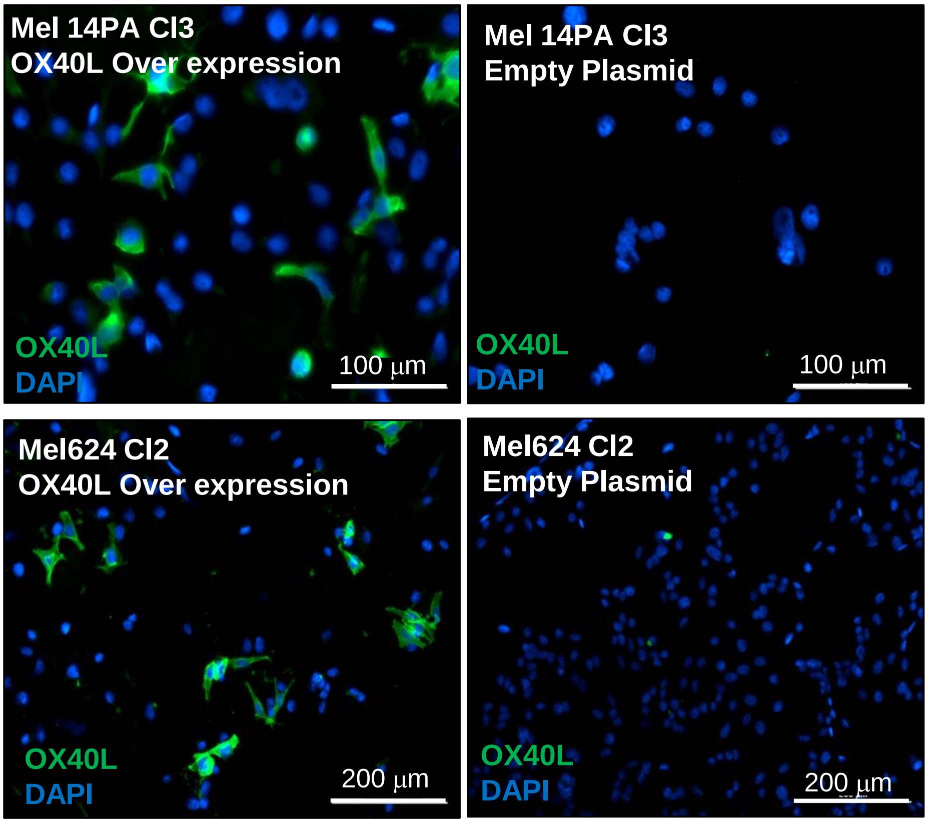

D

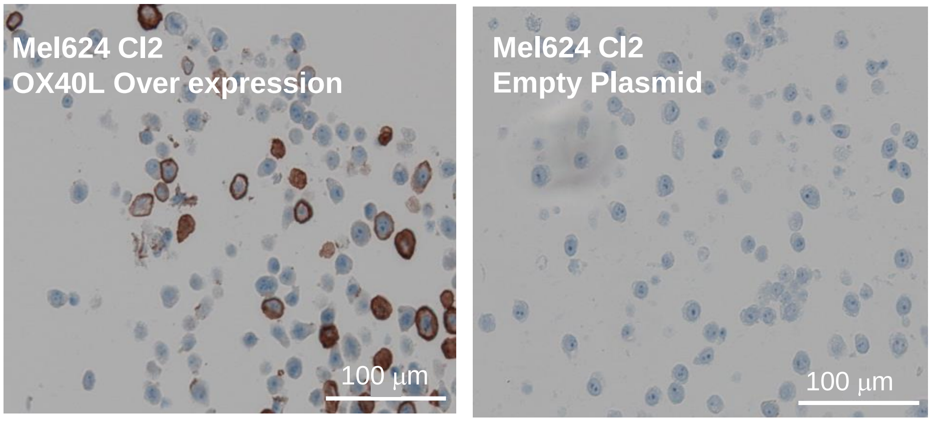

E

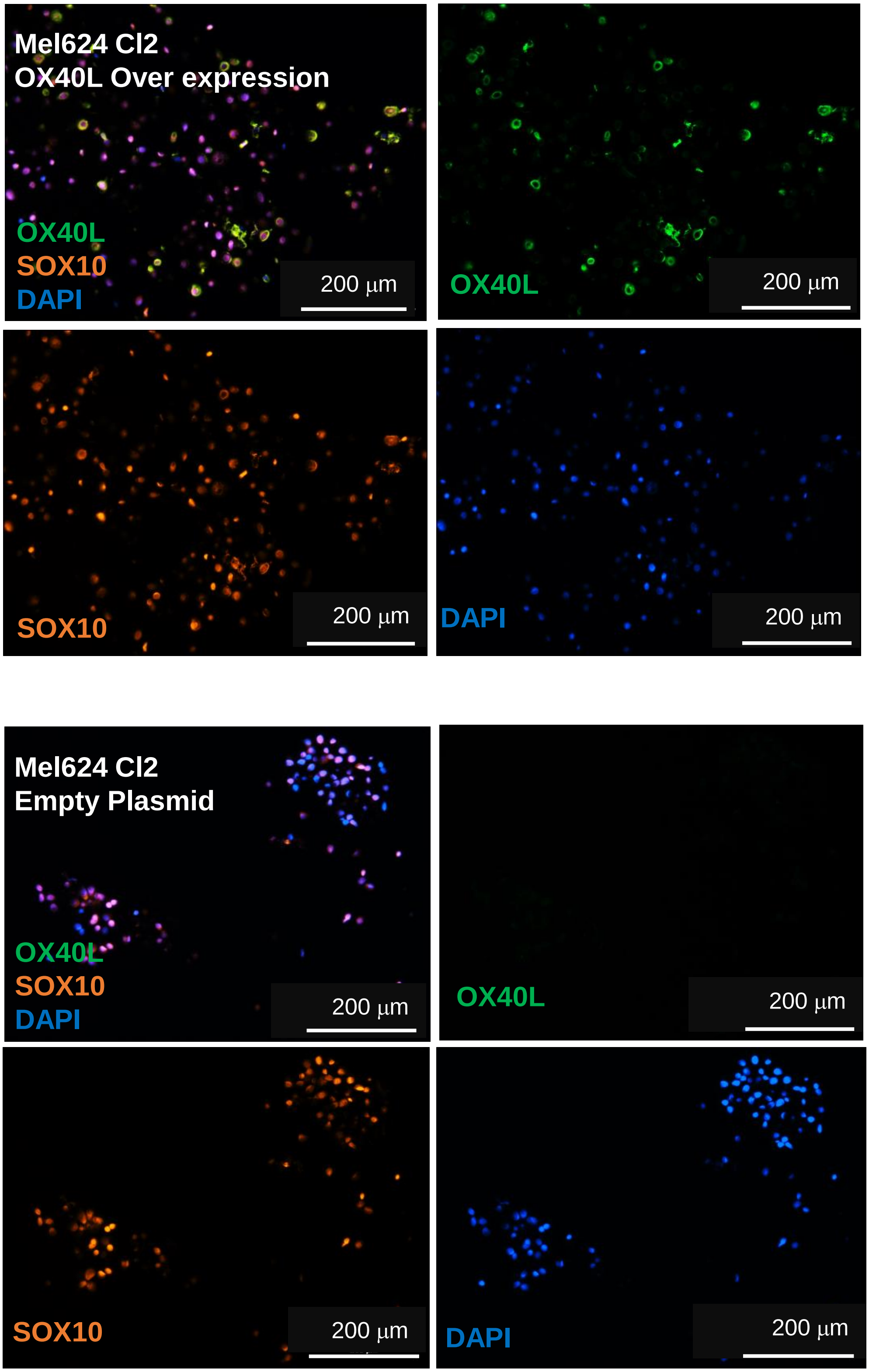

Mel624 Cl2
OX40L Over expression
OX40L
SOX10
DAPI
200 μm
OX40L
200 μm
SOX10
200 μm
DAPI
200 μm
Mel624 Cl2
Empty Plasmid
OX40L
SOX10
DAPI
200 μm
OX40L
200 μm
SOX10
200 μm
DAPI
200 μm

F

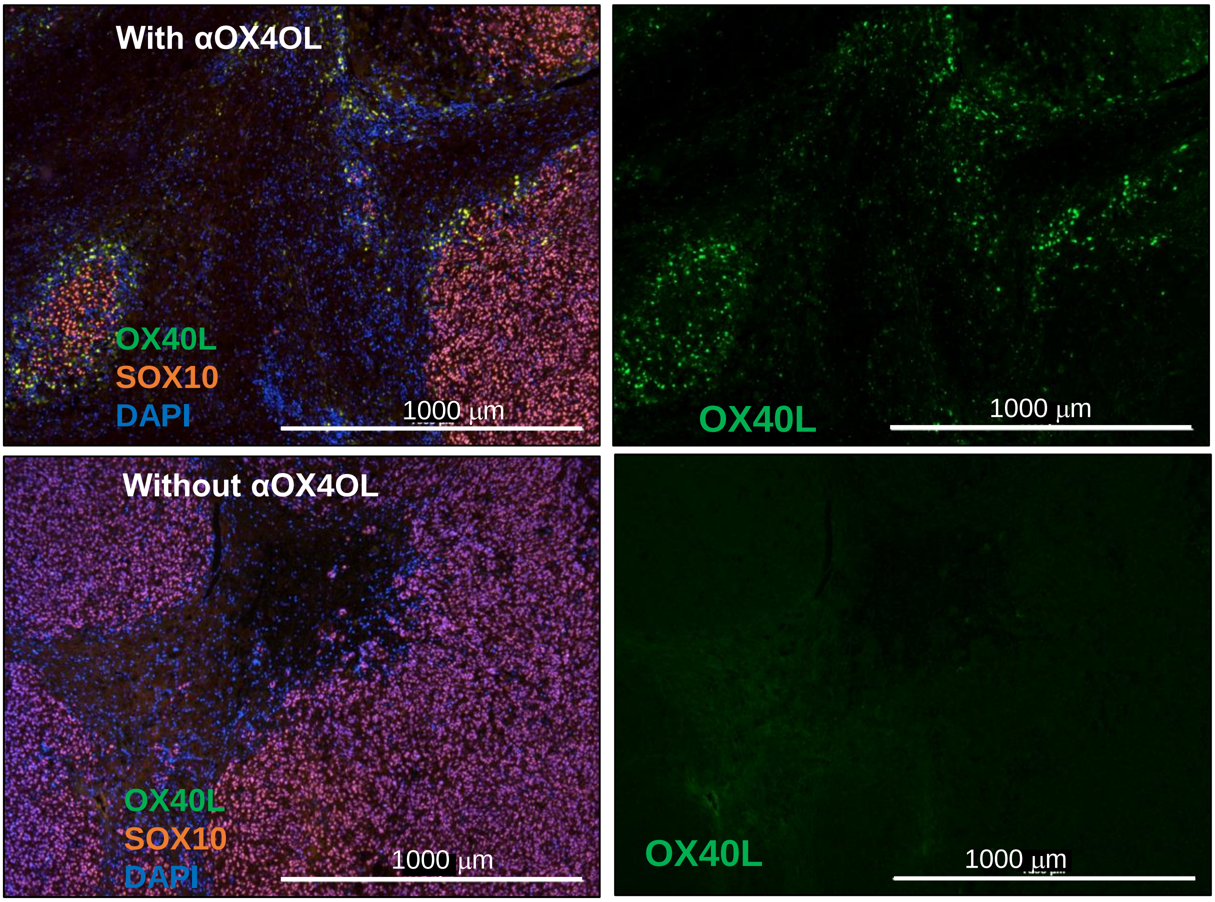
With αOX4OL
OX40L
SOX10
DAPI
1000 µm
OX40L
1000 µm
Without αOX4OL
OX40L
SOX10
DAPI
1000 µm
OX40L
1000 µm

Figure S7. **Validation of anti-OX40L antibody specificity**

(A) Quantitative RT-PCR analysis demonstrating high OX40L (TNFSF4) mRNA expression in stably transfected melanoma cell clones (Mel624 Cl2 and Mel14PA Cl3) compared with parental and empty-vector control cell lines. Expression levels were normalized to GAPDH and are shown as $2^{-\Delta Ct} \times 10^4$.

(B) Western blot analysis of whole-cell lysates showing robust OX40L protein expression in OX40L-transfected melanoma cell clones, with no detectable signal in empty-vector controls. β-Actin was used as a loading control.

(C) Immunocytochemistry performed on melanoma cell clones grown on glass coverslips demonstrating strong OX40L-specific staining in transfected clones and absence of staining in negative controls.

(D) Chromogenic immunohistochemistry of formalin-fixed, paraffin-embedded (FFPE) cell blocks derived from OX40L-transfected and control melanoma cell lines, showing specific membrane-associated OX40L staining exclusively in transfected clones.

(E) Immunofluorescence staining of FFPE cell block sections using the same reagents and amplification protocol as applied to tumor tissue analyses, demonstrating specific OX40L signal in transfected clones but not in negative controls, while nuclear (DAPI) and SOX10 staining were comparable across conditions.

(F) Immunofluorescence analysis of consecutive melanoma FFPE tissue sections processed in parallel with or without primary anti-OX40L antibody, demonstrating specific signal in the presence of the primary antibody and minimal background from secondary reagents alone.
